# Modeling the Evolution of Beliefs Using an Attentional Focus Mechanism

**DOI:** 10.1371/journal.pcbi.1004558

**Published:** 2015-10-23

**Authors:** Dimitrije Marković, Jan Gläscher, Peter Bossaerts, John O’Doherty, Stefan J. Kiebel

**Affiliations:** 1 Department of Psychology, Technical University Dresden, Dresden, Germany; 2 Max Planck Institute for Human Cognitive and Brain Sciences, Leipzig, Germany; 3 Institute for Systems Neuroscience, University Medical Center Hamburg-Eppendorf, Hamburg, Germany; 4 Division of Humanities and Social Sciences, California Institute of Technology, Pasadena, California, United States of America; 5 Department of Finance, University of Utah, Salt Lake City, United States of America; 6 Computation and Neural Systems, California Institute of Technology, Pasadena, California, United States of America; 7 Trinity College Institute of Neuroscience, Trinity College Dublin, Dublin, Ireland; Technische Universitat Chemnitz, GERMANY

## Abstract

For making decisions in everyday life we often have first to infer the set of environmental features that are relevant for the current task. Here we investigated the computational mechanisms underlying the evolution of beliefs about the relevance of environmental features in a dynamical and noisy environment. For this purpose we designed a probabilistic Wisconsin card sorting task (WCST) with belief solicitation, in which subjects were presented with stimuli composed of multiple visual features. At each moment in time a particular feature was relevant for obtaining reward, and participants had to infer which feature was relevant and report their beliefs accordingly. To test the hypothesis that attentional focus modulates the belief update process, we derived and fitted several probabilistic and non-probabilistic behavioral models, which either incorporate a dynamical model of attentional focus, in the form of a hierarchical winner-take-all neuronal network, or a diffusive model, without attention-like features. We used Bayesian model selection to identify the most likely generative model of subjects’ behavior and found that attention-like features in the behavioral model are essential for explaining subjects’ responses. Furthermore, we demonstrate a method for integrating both connectionist and Bayesian models of decision making within a single framework that allowed us to infer hidden belief processes of human subjects.

## Author Summary

When making decisions in our everyday life (*e*.*g*. where to eat) we first have to identify a set of environmental features that are relevant for the decision (e.g. the distance to the place, current time or the price). Although we are able to make such inferences almost effortlessly, this type of problems is computationally challenging, as we live in a complex environment that constantly changes and contains an immense number of features. Here we investigated the question of how the human brain solves this computational challenge. In particular, we designed a new experimental paradigm and derived novel behavioral models to test the hypothesis that attention modulates the formation of beliefs about the relevance of several environmental features. As each behavioral model accounted for a different hypothesis about the underlying computational mechanism we compared them in their ability to explain the measured behavior of human subjects performing the experimental task. The model comparison indicates that an attentional-focus mechanism is a key feature of behavioral models that accurately replicate subjects’ behavior. These findings suggest that the evolution of beliefs is modulated by a competitive attractor dynamics that forms prior expectation about future outcomes. Hence, the findings provide interesting and novel insights into the computational mechanisms underlying human behavior when making decisions in complex environments.

## Introduction

A typical problem that humans encounter, in our complex environment, is to identify those environmental features that are relevant for achieving a desired outcome in a given task. This is computationally difficult because the real-world environment displays a large number of environmental features. In addition, the relevance of the features can change over time and the observations do not always reflect the relevance of specific features. For example, to increase the chance of catching a fish, a fisherman has to consider various features (*e*.*g*. time of the day, lightening conditions, water transparency, *etc*.). Depending on the fishing place (*e*.*g*. pond, lake, or river) only some of these features will be relevant. To perfectly solve such tasks all possible features should be taken into account simultaneously. However, due to an apparent limitation in their cognitive resources, humans dynamically attend only to the most relevant environmental features when deciding what action to pursue [1,2]. Our goal here is to develop a computational model to analyze behavioral data and understand better how attention modulates the update of beliefs about the relevance of features in such complex environments.

An ideal test bed to address these questions is the Wisconsin card sorting task (WCST), as it provides an experimental environment with multiple visual features, in which at any moment of time only a single feature is relevant for correctly solving the task. The WCST was originally designed to test for the damage or dysfunction of the prefrontal cortex, which regulates executive functions [3–6]. More recently it was employed in various behavioral models as a paradigm with which one can investigate computational mechanisms of higher cognitive functions [7].

Here we will focus on the computational mechanisms that underlie update of beliefs about the relevance of various visual features. However, inferring the hidden belief states of subjects performing the standard WCST is difficult, as the only expression of an internal, multidimensional belief space are the behavioral choices [1,8–10]. To address this issue we designed a probabilistic variant of WCST in which we solicited subjects’ beliefs [11], that is, we requested from subjects to bet an amount of money proportionally to their beliefs about the relevance of each visual feature. Importantly, various sources of uncertainty made the environment of WCST probabilistic and made the task more difficult, thus allowing us to measure smooth belief trajectories that evolve over single trials. This fine-grained measure provides more direct access to subjects’ hidden belief states and thus allowed for improved inference, compared to the standard WCST. Using this novel variant of the WCST, we were able to develop a probabilistic model for the analysis of behavioral data to provide novel insights into the hidden learning mechanism, which drives human behavior [12–14].

Previous computational models for the WCST can be divided into three groups based on the assumed computational principle that were used to capture human behavior and cognition: (i) functional cognitive models [10], which are motivated by algorithmic properties of the task; (ii) connectionist models [9,15–19], which are motivated by the evidence that the brain is an active and distributed system that constantly generates hypotheses about its environment and tests for their validity [20–25]; and probabilistic Bayesian models [1], which further assume that the brain combines prior knowledge and present sensory information based on their relative precision, that is, in a Bayes-optimal manner [26–32].

The classical connectionist approach provides an elegant framework for defining attention formation in a distributed and dynamical manner. A potential limitation is that one requires additional and rather ad-hoc assumptions to describe the interaction of prediction errors with internal dynamics of beliefs. This issue can be addressed by the Bayesian approach which provides a framework for defining optimal interaction between prediction errors and current belief states. Furthermore, the Bayesian framework provides a computational account of attention [33–36], which the connectionist approach lacks. Here we build upon these past views of attention within the Bayesian framework, with an attentional focus mechanism that relies on competitive and self-organized dynamical principles that guide spontaneous formation of attention. We will fuse the winner-take-all (WTA) dynamics [37–43] with a Bayesian formalism of decision making.

With this combined approach we can investigate, at the same time, the influence of attention and the influence of probabilistic aspects of the environment on the evolution of beliefs during decision making. In addition, this framework allows us to relate our investigation to previous findings of a presumed hierarchical representation in the brain [12,14,44–48]. Importantly, the introduction of such an attentional focus mechanism within a Bayesian framework takes the model away from the rational Bayesian observer that is fully informed about the structure of the probabilistic WCST and which updates beliefs about all features independent of their relevance. However, we expect an attentional focus mechanism to provide a better account for experimentally observed human behavior.

To test whether subjects’ behavior reflects the assumption that the update of beliefs is modulated by attentional focus we compared multiple variants of the behavioral models, both with and without an attentional focus mechanism, in their ability to generate behavioral data. In particular, we used a recently described meta-Bayesian approach, the so-called ‘Observing the observer’ (OTO) framework to infer the hidden belief states and their influence on behavioral responses of human subjects [49,50]. Importantly, using the OTO framework enabled us to put perception and action (i.e., subjects’ responses) into a single behavioral model and to compare various variants of both perceptual and response models. Each variant of the perceptual model tested for different assumptions about the mechanisms that underlie the update of beliefs. Similarly different variants of the response model tested for evidence regarding sub-optimality in human decision making, caused by a potentially stochastic representation of posterior beliefs in the brain [51–53].

In what follows, we will first describe the experimental paradigm, briefly introduce the OTO framework, and derive the update equations of several variants of the behavioral models. Then we will describe the data analysis technique that relies on Bayesian model selection using a random effects metric [54,55], and present the results of the analysis that we performed on a behavioral, multi-subject, data set obtained from a probabilistic WCST paradigm. In the last section of the article we discuss the relevance of the proposed attentional-focus mechanism and its relation to past works.

## Methods

In this section we will first describe the experimental task, a probabilistic Wisconsin card sorting task with belief solicitation. Afterwards, we will give a brief description of the OTO framework ‘Observing the observer’ [49] and we will introduce the variants of perceptual and response models that we used to model the update of the hidden belief states and the corresponding solicited responses. Finally, we will outline the methods that we applied to estimate the posterior distribution of model parameters and the corresponding model evidence, which we used to perform Bayesian model comparison.

### Ethics statement

The experiment was approved by the Caltech Institutional Review Board and all subjects gave informed consent before participating in the study.

### Probabilistic Wisconsin card sorting task

We designed the experimental task with the aim to access the hidden belief states of the subjects. For this purpose we instructed the subjects to infer, by observing a series of an experimenter's choices, which one of the three different visual features is relevant for the current choice, and to report their beliefs about the relevance of each of the features. Participants in the experiment were all healthy volunteers recruited from the Caltech student population.

**Fig 1:**
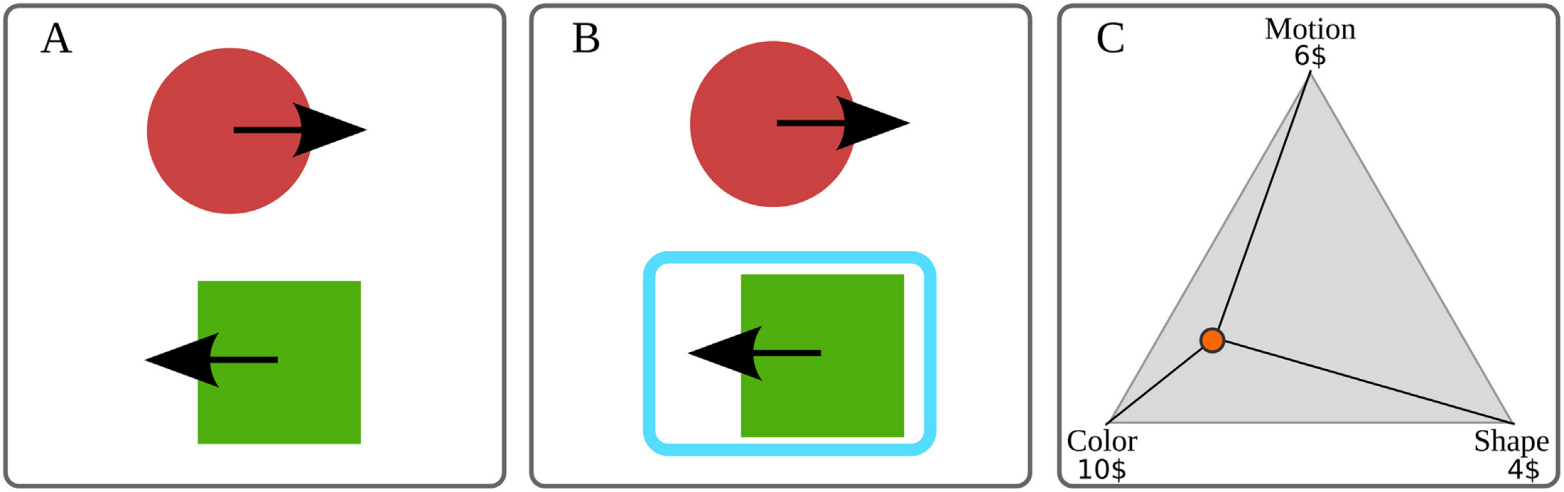
Experimental design. A trial consists of three subsequent steps: (A) The visual stimuli shown in a single trial as two cards. Note that each of the three visual features (color, shape, and motion) has two exemplars (e.g red and green for color) which are assigned either to the top or to the bottom card. (B) The experimenter selects one of the cards, here shown as a blue rectangle. (C) The subject distributes 20$ over three visual features by moving a cursor (red circle) within a triangle. The closer the cursor was to one of the corners of the triangle, the more money was assigned to the corresponding visual feature.

The visual stimuli that we presented to subjects consisted of a pair of cards (top and bottom), where each card contained three visual features (color, motion, shape). In turn, each visual feature was represented by one of the two possible exemplars (red-green, left-right, circle-square). As each card had to contain a distinct exemplar, there were eight distinct configurations of card pairs. Thus at each experimental trial the visual stimulus was randomly selected from one of the eight configurations (e.g., a red right-moving circle and green left-moving square; see Fig 1A).

Each out of *n* = 22 pre-trained subjects (14 male and 8 female) was exposed to an experimental session divided into six blocks consisting of *T* = 40 trials each. In three randomly selected blocks the relevant feature remained fixed (no-switch condition), whereas in the other three blocks the relevant feature would change with a probability *p* = 0.35 (switch condition). After each switch the relevant feature would remain constant for 8 trials before another switch could occur. Importantly, to make the otherwise quite simple task more difficult for healthy subjects we introduced observation uncertainty: the experimenter would select a wrong card (a card not containing the relevant exemplar) with probability *ε* = 0.2 in the no-switch condition, and with probability *ε* = 0.3 in the switch condition. The error rate *ε* was set to values that induced the most distinct behavioral responses between two experimental conditions, while rendering the switch condition informative enough to induce betting responses in subjects.

At the beginning of each experimental block we informed the subjects about the block type, but we did not inform them about the exact values of the error rates ε or switch probabilities; they had to infer these probabilities during the training phase. Each subject went through three training sessions, where each subsequent session slightly increased the difficulty of the task in the following manner: In the first session subjects were exposed to a no switch environment with error rate of experimenters choices set to zero. In the second session the switches in the selection rule where announced with error rate still being set at zero. The third session consisted of the no-switch environment with *ε* = 0.2. Afterwards, we explained to subjects the condition in the final switch environment with non-zero error rate.

During a single trial subjects were first exposed to one of the eight possible visual stimuli (see Fig 1A). After one second the presentation program would select a card containing the relevant exemplar with probability 1 − *ε* (see Fig 1B). After observing the selected choice for 5 seconds subjects had a 4 second period to respond by distributing 20$ on the three visual features depending on their belief about the relevance of each feature for the selection process. The response was generated by moving a cursor within a triangle presented on the screen (see Fig 1C). The closer the cursor was to one of the corners of the triangle the more money was assigned to the corresponding visual feature. Importantly, subjects were told that at the end of the experiment a single trial will be randomly selected and that subjects will gain the amount of money that they assigned to the relevant feature in that trial. This ensured that participants were motivated to provide an accurate rendering of their beliefs over the features.

**Fig 2:**
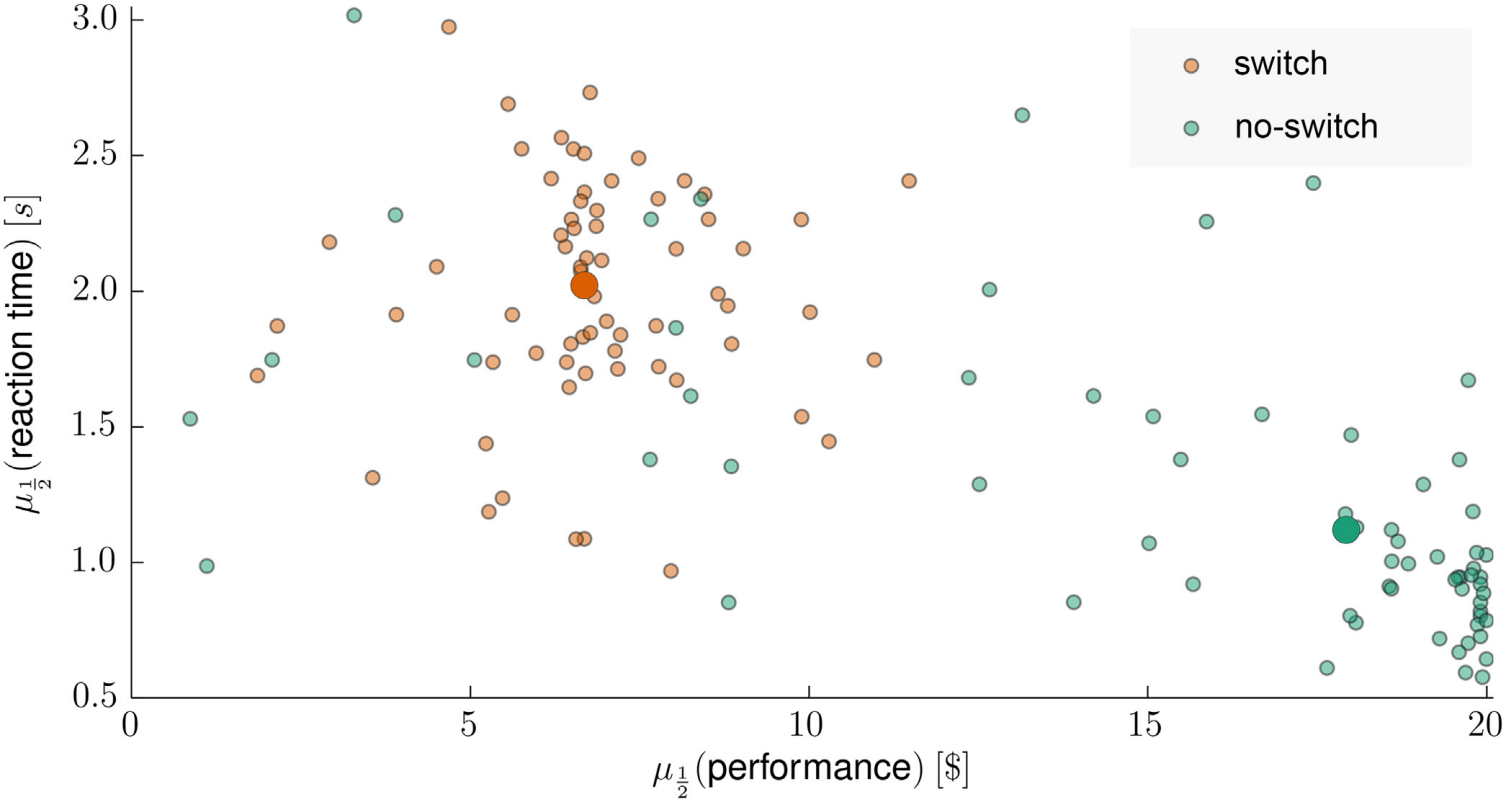
Reaction times and task performance. Median reaction time plotted against median performance of 22 subjects for each of three experimental blocks of the switch (orange circles) and no-switch condition (green circles). The two large circles denote the median values across all experimental blocks within the two experimental conditions. We defined the median performance as the median money gain within an experimental block, that is, the median amount of money assigned to the truly relevant visual feature within an experimental block.

For clarification of the task we present at this point some of the key behavioral results (see Fig 2). We quantified the performance of subjects as the median amount of their money bets on a truly relevant visual feature over an experimental block. The maximal performance would correspond to betting the full amount of 20$ to the truly relevant feature at each trial. As expected, the median of subjects’ performance was higher during the no-switch condition (Kruskal-Wallis test, *p* <10^−14^), whereas the median reaction times were lower (Kruskal-Wallis test, *p* <10^−12^) during the same experimental condition which reflects the increased difficulty of the switch condition.

### ‘Observing the Observer’ framework

**Fig 3:**
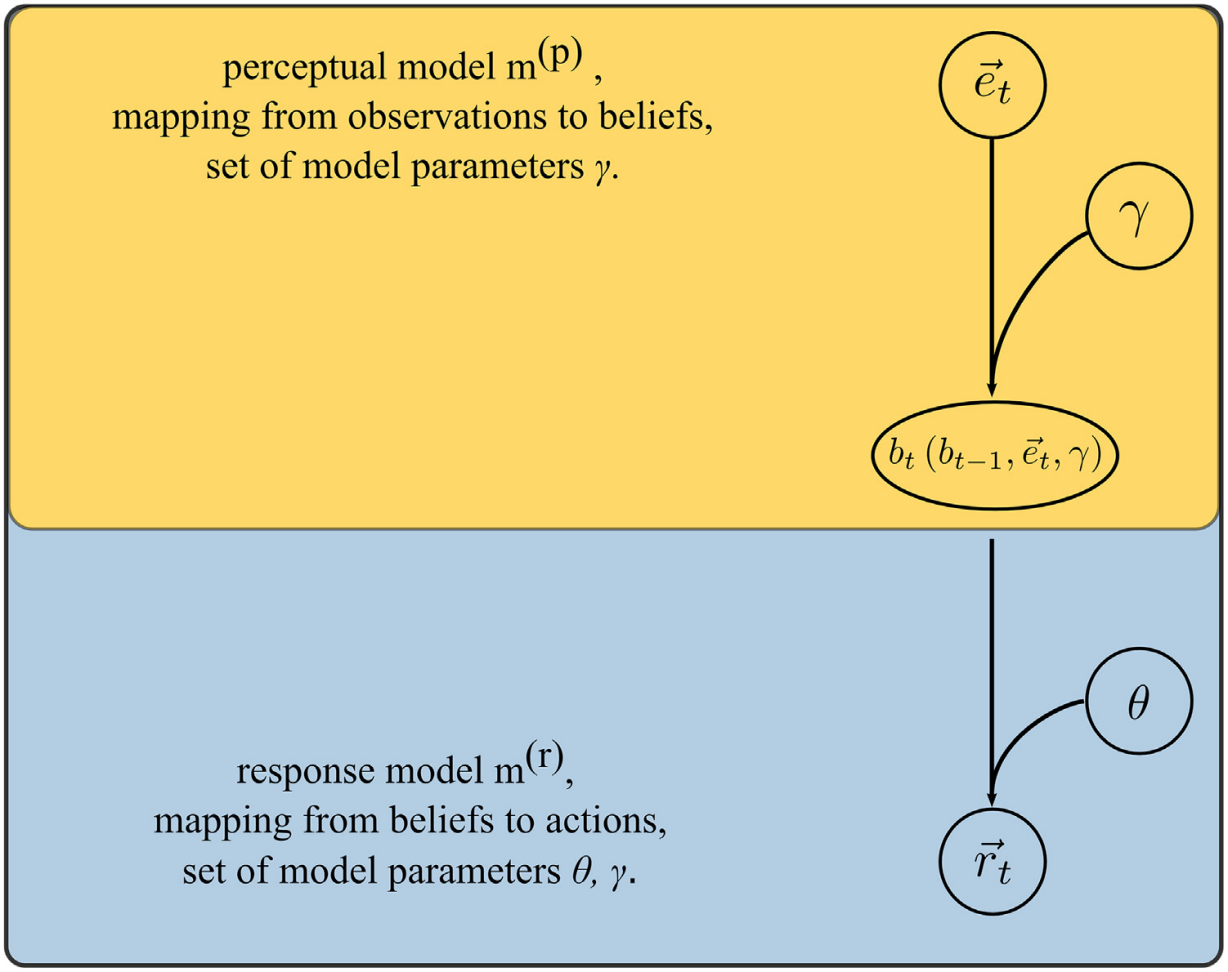
Schematic of implicit generative model as formulated under the Observing-the-observer framework. The full generative model consists of a combined perceptual (orange box) and response model (blue box). The perceptual part of the generative model defines the mapping from current observations ${\vec{e}}_{t}$, past beliefs *b*_*t*−1_, and a set of model parameters *γ*, onto current beliefs *b*_*t*_. The response part of the generative model defines the mapping from current beliefs *b*_*t*_ and a set of model parameters *θ*, onto responses ${\vec{r}}_{t}$. Figure adapted from [49].

Our goal is to infer, from the behavioral data, the hidden belief states of each subject that are conditioned on the past sequence of visual stimuli and experimenter choices. By deriving an adequate mapping of observations onto internal belief states (the perceptual model) and the mapping of the internal belief states onto desired responses (response model), we can define a generative model of the whole observation-response process [49,50] as (see Fig 3 for a graphical representation):

$$p\left({\vec{r}}_{t},\gamma ,\theta |{\vec{e}}_{t},m^{\left(p\right)},m^{\left(r\right)}\right)=p\left({\vec{r}}_{t}|b_{t}\left(b_{t-1},{\vec{e}}_{t},\gamma \right),\;\theta ,m^{\left(p\right)},\;m^{\left(r\right)}\right)p\left(\gamma ,\theta |m^{\left(r\right)},m^{\left(p\right)}\right),$$ (1)

where $p\left({\vec{r}}_{t}|b_{t}\left(b_{t-1},{\vec{e}}_{t},\gamma \right),\;\theta ,m^{\left(p\right)},m^{\left(r\right)}\right)$ denotes the probability of observing a response ${\vec{r}}_{t}$ given the hidden belief states *b*_*t*_ (that depend on past beliefs, current sensory observations ${\vec{e}}_{t},$ and a set *γ* of free parameters of the perceptual model *m* ^(*p*)^) and a set *θ* of free parameters of the response model *m* ^(r)^. The last term *p*(*γ*, *θ*|*m* ^(r)^, *m* ^(*p*)^) in Eq (1) denotes a prior distribution over the space of free parameters.

Thus, to infer the hidden belief states of a subject we have to invert the generative model (Eq (1)) for the given set of behavioral responses *r*_1…*t*_ and sensory stimuli *e*_1…*t*_, and compute the posterior distribution over the model parameters

$$p\left(\gamma ,\theta |e_{1\ldots t},r_{1\ldots t}\right)=\;\frac{p(\gamma ,\theta )\prod_{k=1}^{t}p\left({\vec{r}}_{k}|\gamma ,\theta ,e_{1\ldots k}\right)}{p\left(r_{1\ldots t}|e_{1\ldots t}\right)},$$ (2)

where we omitted *m* ^(*r*)^, *m* ^(*p*)^ for better readability. Knowing the posterior distribution one can either compute the most likely belief state at trial *t* as $b_{t}\left(\hat{\gamma }\right)—$ where $\hat{\gamma }$ denotes the mode of the posterior—or an expected belief state at trial t, as ${\bar{b}}_{t}=E_{p\left(\;\gamma |e_{1\ldots t},r_{1\ldots t}\right)}[b_{t}(\gamma )]$.

To test the hypothesis that subjects focus their attention on a subset of environmental features when updating their beliefs about the features' relevance, it is essential to compare multiple models in their ability to replicate the behavioral data and select the most appropriate model. Bayesian model comparison uses model evidence, that is, marginal likelihood *p*(*r*_1…*t*_|*e*_1…*t*_), to estimate the probability that a specific model has generated the data. The advantage of such a procedure, compared to standard goodness of fit approaches, is that more complex models are penalized automatically. The model evidence, for any pair of perceptual and response models, is given as

$$p\left(r_{1\ldots t}|e_{1\ldots t},\;m^{\left(p\right)},m^{\left(r\right)}\right)=\;\int^{\text{​}}d\gamma d\theta p(\gamma ,\theta )\prod_{k=1}^{t}p\left(r_{k},\;|\gamma ,\theta ,e_{1\ldots k},m^{\left(p\right)},m^{\left(r\right)}\right).$$ (3)

To estimate the model evidence and obtain the posterior distribution over model parameters *p*(*γ*,*θ*|*e*_1…*t*_, *r*_1…*t*_) any approximate inference scheme can be applied. In particular, Daunizeau *et*. *al*. [49,50] proposed the use of a variational scheme where the model log-evidence is approximated with the variational free-energy and the posterior distribution over the model parameters is selected as the maximizer of the free-energy obtained through variational calculus. However, this method requires the computation of the gradients of the log-joint probability distributions (natural logarithm of the joint probability distribution given in Eq (1)), which in our case are not obtainable analytically as the derivatives affect the parameters of the non-linear equations of the belief process. Furthermore, a small change in the parameters of the update equations of beliefs (Eq (11), see below) can have a large influence on the shape of the trajectory, thus the log-joint probability distribution can be ill-conditioned with respect to model parameters. Therefore, even if the gradient, with respect to model parameters, would be computable at every point of the trajectory, a gradient ascent method would have difficulties to converge to a global mode of the joint probability distribution, as the underlying landscape might have a multimodal, non-linear, and non-convex structure.

Thus, we use a numerical gradient-free scheme to find the mode of the log-joint probability distribution and apply a numerical method to compute the Hessian matrix at that mode [56–58]. With the obtained values of the mode and the Hessian we compute the Laplace approximation to the model evidence [59]. We will discuss the specifics of the numerical estimates in the final subsection of the methods. In what follows we will first introduce the behavioral models.

#### Perceptual model

To derive the perceptual model, which maps sensory cues onto beliefs we followed previous accounts in making three important assumptions [60–62]. First, we will assume that subjects combine prior beliefs and sensory information in a Bayes optimal fashion (Bayesian observer assumption). Note that this assumption will later be relaxed to obtain a non-Bayesian approximation to the update equations. Second, we assume that the update of beliefs can be represented as a Markov process, that is, future belief states depend only on the present beliefs. Third, we will assume that subjects perform counterfactual inference [35], that is, they try to infer which of the several hypothesis (explanations of experimenter’s choices) is currently correct. A single hypothesis would correspond to saying that the experimenter selects cards containing a specific exemplar (e.g. color red). As each visual feature has two exemplars (red-green color, leftward-rightward motion, and round-square shape), there are in total six hypotheses.

Starting with these three assumptions we will define a generative model of the sensory observations in the form of a hierarchical state space model [63], that captures the dynamics of the transient probability that one of the six possible selection rules is currently active. Inversion of the generative model will provide us with the required mapping from sensory cues onto posterior probability about the correctness of each hypothesis, that is, the posterior beliefs about the relevance of different visual features and exemplars.

However, to specify the structure of the hierarchical generative model, a few additional assumptions are required. First, we can assume that the probability *p*(*H*_*t*_) of hypothesis *H*_*t*_ being correct is represented in a factorized from, that is, *p*(*H*_*t*_) equals to the product of the probability *p*(*F*_*t*_) that one of the visual features *F*_*t*_ is currently relevant and of the conditional probability *P*(*E*_*t*_|*F*_*t*_) that one of the two exemplars *E*_*t*_ is currently relevant (given the fact that the corresponding visual feature *F*_*t*_ is relevant for the selection process). Alternatively, we can assume that only the probability *p*(*H*_*t*_) of hypothesis *H*_*t*_ being correct is explicitly represented and that the marginal probability *p*(*F*_*t*_) is computed only implicitly via the integration of corresponding beliefs.

Depending on the starting assumption one will end up with slightly different structure of the corresponding hierarchical generative model. Here we will describe in detail only the generative model based on the assumption that only the joint hypothesis probability *p*(*H*_*t*_) is explicitly represented and actively updated within the belief space. The reason for this is that model comparisons (see below) suggest that such representation better captures subject behavior. Nevertheless, the detailed derivation and the analysis of the behavioral data based on the alternative assumption, mentioned above, are provided in the supplementary material (S1 Text).

**Fig 4:**
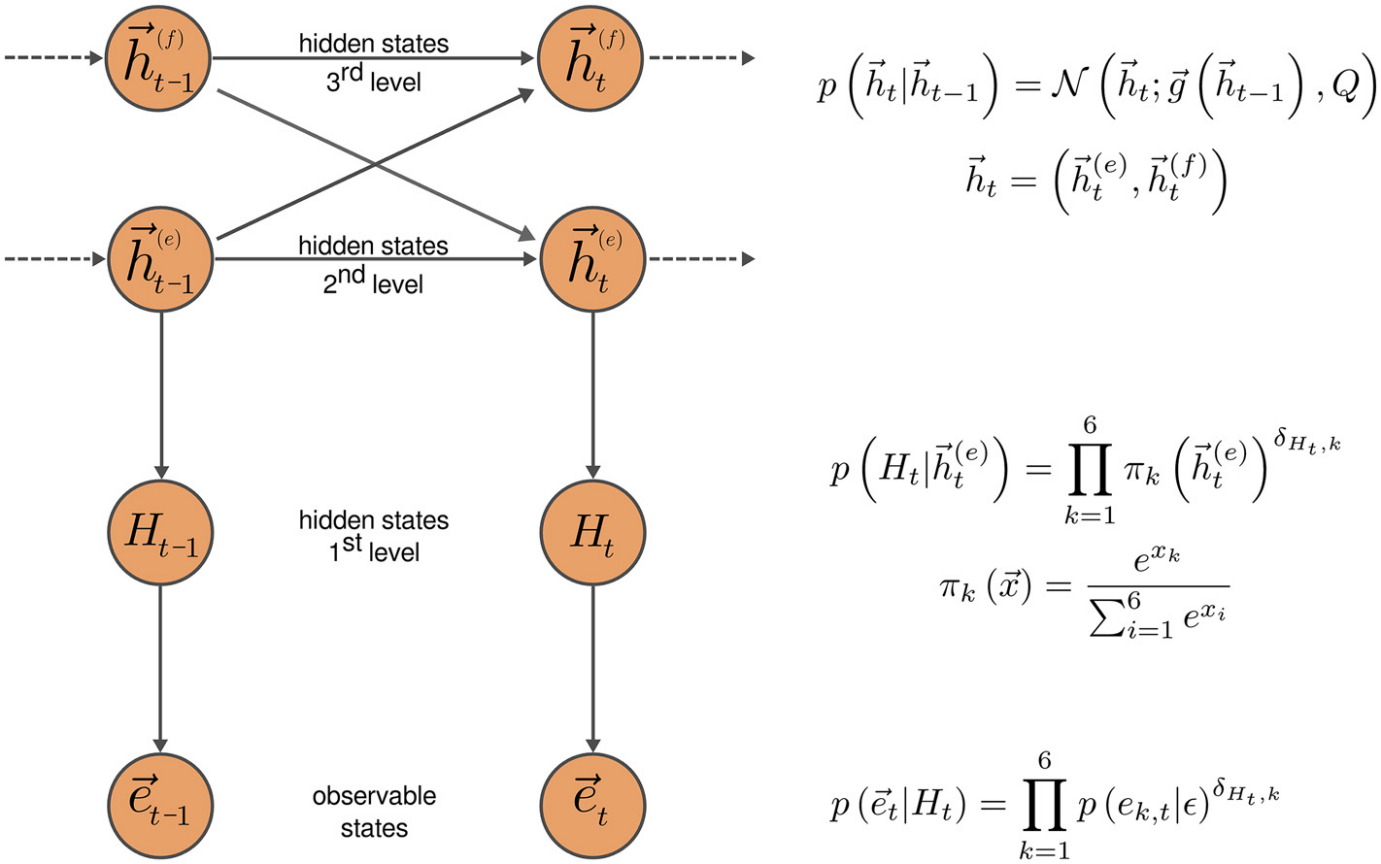
A graphical representation of the hierarchical generative model of percepts. The highest 3^rd^ level hierarchy describes the dynamics of the three dimensional state space vector $\;{\vec{h}}_{t}^{\left(f\right)}$ that encodes the relevance of the three visual features. Similarly, the 2^nd^ level of the hierarchy describes the dynamics of the six dimensional state space vector ${\vec{h}}_{t}^{\left(e\right)}$ that encodes the relevance for the selection process of the six exemplar-feature pairs. The functional form of the state transition probability $p\left({\vec{h}}_{t}|{\vec{h}}_{t-1}\right)$ is shown on the right hand side of the plot. The 1^st^ level of the hierarchy encodes the currently active selection rule, that is, a currently correct hypothesis *H*_*t*_, where the currently correct hypothesis is drawn from a conditional probability $p\left(H_{t}|{\vec{h}}_{t}^{\left(e\right)}\right)$ shown on the right hand side of the plot. Finally, the observable states are denoted with the six dimensional vector ${\vec{e}}_{t}$, that encodes currently selected exemplars. On the right hand side of the plot we show the conditional probability $p\text{(}{\vec{e}}_{t}|H_{t})$ of selecting the *k* th exemplar given the active selection rule *H*_*t*_. For details, see the Eqs (4) to (7) and the accompanying text.

Here we will define the generative model as a three-level hierarchy (see Fig 4 for graphical representation): (i) the 1^st^ level of the hierarchy encodes the hidden selection rule, that is, the currently correct hypothesis *H*_*t*_ (see Eq (5)); (ii) the 2^nd^ level of the hierarchy encodes the probability, in the form of a state space vector ${\vec{h}}_{t}^{\left(e\right)}$, that each of the possible exemplar-feature pairs is currently relevant for the experimenter’s choices (see Eq (6)), and (iii) the 3^rd^ level of the hierarchy encodes the probability, in the form of the state space vector $\;{\vec{h}}_{t}^{\left(f\right)},$ that each visual feature is currently relevant for the experimenter’s choices (see Eq (7)).

Assuming that the *k* th hypothesis is the correct one (*k* ∈ {1,…,6}), the corresponding exemplar will be selected with probability 1 − *ε*, where *ε* denotes the error rate of experimenter’s choices. We will encode the experimenter’s choice with a binary vector ${\vec{e}}_{t}\in {\left\{0,1\right\}}^{6}$ whose elements are set to 1 or 0 depending on the presence or absence of the corresponding exemplar on the selected card. Thus, we can write the observation likelihood as

$$p\left({\vec{e}}_{t}|H_{t}\right)=\overset{6}{\underset{k=1}{\prod }}p{\left(e_{k,t}|\varepsilon \right)}^{\delta_{H_{t},k}};\;\;\;\;p\left(e_{k,t}|\varepsilon \right)=\;{\left(1-\varepsilon \right)}^{e_{k,t}}\varepsilon^{1-e_{k,t}},$$ (4)

where $\;\delta_{H_{t},k}$ denotes Kronecker's delta and *e*_*k*_,_*t*_ denotes the *k*th component of ${\vec{e}}_{t}.$

At the 1^st^ (lowest) level of the hierarchy, we defined the probability that a hypothesis *H*_*t*_ ∈ {1,…,6} is the correct one as a categorical probability distribution

$$p\left(H_{t}|{\vec{h}}_{t}\right)=\;\overset{6}{\underset{k=1}{\prod }}\pi_{k}{\left({\vec{h}}_{t}^{\left(e\right)}\right)}^{\delta_{H_{t},k}},$$ (5)

where the $\pi_{k}\left({\vec{h}}_{t}^{\left(e\right)}\right)$ denotes the probability of the *k* th hypothesis. These probabilities are encoded at the 2^nd^ level of the hierarchy (see Fig 4) with the real valued vector ${\vec{h}}_{t}^{\left(e\right)}\in {\mathbb{R}}^{6}$, where we defined the mapping to the space of categorical probabilities as the softmax transform

$$\pi_{k}\left({\vec{h}}_{t}^{\left(e\right)}\right)=\;\frac{e^{h_{k,t}^{\left(e\right)}}}{\sum_{j=1}^{6}e^{h_{j,t}^{\left(e\right)}}}.$$

To incorporate an attention-like mechanism within the perceptual model, we make the state transition of ${\vec{h}}_{t}^{\left(e\right)}$ to follow a winner-take all (WTA) dynamics. We used this type of dynamics for three reasons:

1. The WTA dynamics is characterized by a set of stable fixed points that can be arranged in such a way that at each fixed point only one component of ${\vec{h}}_{t}^{\left(e\right)}$ is set to a high value (which encodes a high relevance of the corresponding exemplar), while all other components have low values. Such attractor state captures the structure of the WCST environment, in which at any moment in time only one exemplar-feature pair can be relevant.
2. Adding uncorrelated noise to the WTA dynamics mediates the switching between stable attractors. The larger the noise term the more probable is the transition between attractors. Thus, we can use a single parameter that defines the level of noise in the WTA dynamics to capture different experimental conditions.
3. WTA networks were successfully used before as a hierarchical neural model of higher cognitive functions [8,15,16,18,21,25,64,65], and as a model of attention spontaneously emerging from competitive neural dynamics [66].

Thus, assuming the WTA dynamics, the time evolution of ${\vec{h}}_{t}^{\left(e\right)}$ becomes

$${\vec{h}}_{t+1}^{\left(e\right)}=\;\tau_{e}{\vec{h}}_{t}^{\left(e\right)}+\;\kappa_{e}+W_{lat}^{\left(e\right)}\varphi \left({\vec{h}}_{t}^{\left(e\right)}-\kappa_{e}\right)+W_{dist}^{\left(f\right)}\varphi \left({\vec{h}}_{t}^{\left(f\right)}-\kappa_{f}\right)+{\vec{\omega }}_{t}^{\left(e\right)}.$$ (6)

Here $\varphi \left(x\right)=\frac{1}{1+e^{-x}}$ and $\varphi \left(\vec{y}\right)=\left(\varphi \left(y_{1}\right),\cdots ,\varphi \left(y_{n}\right)\right);$ ${\vec{\omega }}_{t}^{\left(e\right)}$ denotes a vector of i.i.d. random variables drawn from normal distribution $N({\vec{\omega }}_{t}^{\left(e\right)};0,\;q_{e}I_{6})$ with zero mean and variance *q*_*e*_; *τ*_*e*_ denotes the time scale of the update equations, and *κ*_*e*_ an additive constant. Importantly, the dynamics of the ${\vec{h}}_{t}^{\left(e\right)}$ is influenced by the state vector ${\vec{h}}_{t}^{\left(f\right)}\in {\mathbb{R}}^{3}$ at the 3^rd^ level of hierarchy, which encodes the relevance of the three visual features (see Fig 4). The time evolution of ${\vec{h}}_{t}^{\left(f\right)}$ is defined by an analogous set of equations

$${\vec{h}}_{t+1}^{\left(f\right)}=\;\tau_{f}{\vec{h}}_{t}^{\left(f\right)}+\;\kappa_{f}+W_{lat}^{\left(f\right)}\varphi \left({\vec{h}}_{t}^{\left(f\right)}-\kappa_{f}\right)+W_{dist}^{\left(e\right)}\varphi \left({\vec{h}}_{t}^{\left(e\right)}-\kappa_{e}\right)+{\vec{\omega }}_{t}^{\left(f\right)}.$$ (7)

Importantly, the connectivity matrices $W_{lat}^{\left(e\right)},W_{lat}^{\left(f\right)}$ denote the inhibitory interactions within levels, which are essential for the realization of attractor dynamics and $W_{dist}^{\left(e\right)},W_{dist}^{\left(f\right)}$ denote the excitatory interactions between the levels of the hierarchy. This allows for integrating the beliefs about hypothesis relevance into beliefs about feature relevance.

In what follows, to simplify the notation, we will merge the state vectors $\;{\vec{h}}_{t}^{\left(e\right)}$ and ${\vec{h}}_{t}^{\left(f\right)}$ into a single state vector ${\vec{h}}_{t}=\left({\vec{h}}_{t}^{\left(e\right)},\;{\vec{h}}_{t}^{\left(f\right)}\right)$ whose update equation is denoted by $\vec{g}\left(\vec{x}\right)$, that is,

$${\vec{h}}_{t+1}=\;\vec{g}\left({\vec{h}}_{t}\right).$$

#### Bayesian inference

Given the observation likelihood Eq (4), hypothesis probability Eq (5) and the transition probabilities Eqs (6) and (7) we write the full generative model as

$$p\left({\vec{e}}_{t},H_{t},{\vec{h}}_{t},{\vec{h}}_{t-1}|e_{1\ldots t-1}\right)=p\left({\vec{e}}_{t}|H_{t}\right)p\left(H_{t}|{\vec{h}}_{t}\right)p({\vec{h}}_{t}|{\vec{h}}_{t-1})p({\vec{h}}_{t-1}|e_{1\ldots t-1}\text{)},$$ (8)

where $e_{1\ldots t-1}=\left({\vec{e}}_{1},\ldots ,{\vec{e}}_{t-1}\right)$ denotes all past observations. As we are interested in obtaining the posterior probability of the hidden states $p\left(H_{t},{\vec{h}}_{t}|e_{1\ldots t}\right)$, we require a compact form of the generative model

$$p\left({\vec{e}}_{t},H_{t},{\vec{h}}_{t}|e_{1\ldots t-1}\right)=\overset{\infty }{\underset{-\infty }{\int }}\ldots \overset{\infty }{\underset{-\infty }{\int }}p\left({\vec{e}}_{t},H_{t},{\vec{h}}_{t},{\vec{h}}_{t-1}|e_{1\ldots t-1}\right)d{\vec{h}}_{t-1}.$$

To obtain this compact form it is necessary to calculate the following integral

$$p({\vec{h}}_{t}|e_{1\ldots t-1}\text{)},=\overset{\infty }{\underset{-\infty }{\int }}\ldots \overset{\infty }{\underset{-\infty }{\int }}p\left({\vec{h}}_{t}|{\vec{h}}_{t-1}\right)p\left({\vec{h}}_{t-1}|e_{1\ldots t-1}\right)d{\vec{h}}_{t}.$$

Assuming that $p\left({\vec{h}}_{t-1}|e_{1\ldots t-1}\right)$ is a normal distribution with mean ${\vec{\mu }}_{t-1}$ and covariance matrix Σ_*t*−1_, we can approximate the integral on the right hand side as

$$p\left({\vec{h}}_{t}|{\vec{e}}_{1\ldots t-1}\right)=N\left({\vec{h}}_{t};\vec{g}\left({\vec{\mu }}_{t-1}\right),\partial_{\vec{h}}\vec{g}{\;\Sigma }_{t-1}\partial_{\vec{h}}{\vec{g}}^{T}+Q\right),\;\;\;Q=q_{e}I_{6}⊕q_{f}I_{3}$$

$$p\left({\vec{h}}_{0}\right)=N\left({\vec{h}}_{0};{\vec{\mu }}_{0},\;{\;\Sigma }_{0}\right),\;\;{\vec{\mu }}_{0}=\left({\vec{\mu }}_{e}^{0},{\vec{\mu }}_{f}^{0}\right)\text{ and }{\;\Sigma }_{0}=\sigma_{e}^{0}I_{6}⊕\sigma_{f}^{0}I_{3},$$

where the approximate predictive distribution $p\left({\vec{h}}_{t-1}|e_{1\ldots t-1}\right)$ is obtained by linearizing $\vec{g}\left(\vec{x}\right)$ around the currently known mean ${\vec{\mu }}_{t-1}$, and where ⊕ denotes direct sum of matrices which constructs a block diagonal matrix from the elements of the sum.

To invert the generative model we apply the variational Bayesian method and the mean-field approximation in which the posterior distribution is approximated by a variational distribution. Thus, we write the posterior probability over the hidden states as a product of approximate posterior distributions, that is

$$p\text{(}H_{t},{\vec{h}}_{t}|{\vec{e}}_{1\ldots t})=q\left(H_{t}\right)q\left({\vec{h}}_{t}\right),$$

where we chose the functional forms of the approximate posteriors as the distribution with maximum entropy given the specified mean and variance. This procedure allows for minimal assumptions about the form of the approximate posterior [46]. Hence, for the posterior probability over the discrete space of hypotheses we selected again a categorical probability

$$q\left(H_{t}\right)=\overset{6}{\underset{k=1}{\prod }}\rho_{t,k}{}^{\delta_{H_{t},k}}\;,$$

whereas for the posterior beliefs about the relevance of exemplars and visual features we selected a multivariate normal distribution

$$q\left({\vec{h}}_{t}\right)=N\left({\vec{h}}_{t};{\vec{\mu }}_{t},\Sigma_{t}\right).$$

Note that in this formulations the posterior belief is fully defined by the tuple of the posterior expectations and the posterior covariance, that is, posterior uncertainty; hence we will denote beliefs as a set $b_{t}=\left\{{\vec{\mu }}_{t},\Sigma_{t}\right\}$.

Following variational calculus, the approximate posterior, given the mean-field approximation, is proportional to the exponential of the variational energy [67]. The variational energies for the given generative model and the above mentioned factorization of approximate posterior are defined as

$$\begin{array}{c} I\left(H_{t}\right)=\overset{\infty }{\underset{-\infty }{\int }}\ldots \overset{\infty }{\underset{-\infty }{\int }}q\left({\vec{h}}_{t}\right)\text{ln}p\left({\vec{e}}_{t},\;H_{t},{\vec{h}}_{t}|{\vec{e}}_{1\ldots t-1}\right)d{\vec{h}}_{t},\\ I\left({\vec{h}}_{t}\right)=\underset{H_{t}\in \{H_{1},\ldots ,\;H_{6}\}}{\sum }q\left(H_{t}\right)\text{ln}p\left({\vec{e}}_{t},H_{t},{\vec{h}}_{t}|{\vec{e}}_{1\ldots t-1}\right). \end{array}$$

To find the dependency of current beliefs *b*_*t*_ on prior beliefs *b*_t-1_ and current observation ${\vec{e}}_{t}$ we used a series of approximations previously described in [46], which we extended to the multidimensional case.

First, to compute *I*(*H*_*t*_), we need to know the beliefs *b*_*t*_, whose computations require knowing $I\left({\vec{h}}_{t}\right)$, which is a functional of *q*(*H*_*t*_), thus leading to a circular problem. We break the circularity by computing *I*(*H*_*t*_) with the expected beliefs $\;\;{\hat{b}}_{t}\;\in \left\{\vec{g}\left({\vec{\mu }}_{t-1}\right),\;\;\partial_{\vec{h}}\vec{g}{\text{ Σ}}_{t-1}\partial_{\vec{h}}{\vec{g}}^{T}+Q\right\}$; hence, we assume that the information about the observation ${\vec{e}}_{t}$ first changes the 1^st^ level of the model’s hierarchy and then propagates to the 2^nd^ and 3^rd^ level. As the exponential of the *I*(*H*_*t*_) has the form of a categorical distribution, one can show with simple algebraic manipulations that

$$\rho_{t,k}=\;\frac{p\left(e_{t,k}|\varepsilon \right)e^{g_{k}({\vec{\mu }}_{t-1})}}{\sum_{j=1}^{6}p\left(e_{t,j}|\varepsilon \right)e^{g_{j}({\vec{\mu }}_{t-1})}}\;.$$ (9)

With the known ${\vec{\rho }}_{t}$ one can compute the $I\left({\vec{h}}_{t}\right)$, where the difficulty is that the variational energy does not have a quadratic form, that is, the exponential of $I\left({\vec{h}}_{t}\right)$ is not a Gaussian distribution. Thus, to obtain a Gaussian form of the approximate posterior we need an additional quadratic approximation to the variational energy

$$\begin{array}{c} \hat{I}\left({\vec{h}}_{t}\right)=I\left(\vec{g}\left({\vec{\mu }}_{t-1}\right)\right)+\partial_{{\vec{h}}_{t}}\;I\left(\vec{g}\left({\vec{\mu }}_{t-1}\right)\right)\left({\vec{h}}_{t}-\vec{g}\left({\vec{\mu }}_{t-1}\right)\right)\\ +\frac{1}{2}{\left({\vec{h}}_{t}-\vec{g}\left({\vec{\mu }}_{t-1}\right)\right)}^{T}\left[\partial_{{\vec{h}}_{t}}^{2}I\left(\vec{g}\left({\vec{\mu }}_{t-1}\right)\right)\right]\left({\vec{h}}_{t}-\vec{g}\left({\vec{\mu }}_{t-1}\right)\right), \end{array}$$

where we made a second order Taylor expansion of $I\left({\vec{h}}_{t}\right)$ around the predictive mean $\vec{g}\left({\vec{\mu }}_{t-1}\right)$, that is, the anticipated position of the posterior expectation. Finally, having the quadratic form we get the posterior mean ${\vec{\mu }}_{t}$ as the argument of the maximum of $\hat{I}\left({\vec{h}}_{t}\right).$ The maximum is obtained with the Newton’s method

$${\vec{\mu }}_{t}=\text{argmax }\hat{I}\left({\vec{h}}_{t}\right)={\vec{h}}_{t}-{\left[\partial_{{\vec{h}}_{t}}^{2}\hat{I}\left({\vec{h}}_{t}\right)\right]}^{-1}\partial_{{\vec{h}}_{t}}\;\hat{I}\left({\vec{h}}_{t}\right).$$ (10)

As Eq (10) is valid for any point ${\vec{h}}_{t}$ of the quadratic function $\hat{I}\left({\vec{h}}_{t}\right)$, we can select again the expansion point $\vec{g}\left({\vec{\mu }}_{t-1}\right)$ as the starting value. In this way we obtain the following update equations for the expected relevance of the hidden states

$$\begin{array}{c} {\vec{\mu }}_{t}=\vec{g}\left({\vec{\mu }}_{t-1}\right)+\Sigma_{t}{\vec{\delta }}_{t},\\ {\vec{\delta }}_{t}=\left({\vec{\rho }}_{t}-\vec{\pi }\left({\vec{g}}^{\left(e\right)}\left({\vec{\mu }}_{t-1}\right)\right),{\vec{0}}_{3}\right), \end{array}$$ (11)

where ${\vec{0}}_{3}$ denotes the three-dimensional zero vector and where the posterior covariance Σ_*t*_ is given as the inverse of the negative Hessian at the expansion point $\vec{g}\left({\vec{\mu }}_{t-1}\right)$, that is,

$$\Sigma_{t}=-{\left[\partial_{{\vec{h}}_{t}}^{2}I\left(\vec{g}\left({\vec{\mu }}_{t-1}\right)\right)\right]}^{-1}.$$

The posterior covariance is updated as

$$\Sigma_{t}=\frac{{\hat{\Sigma }}_{{}_{t-1}}}{I+\;{\hat{\Sigma }}_{{}_{t-1}}Y_{t}};\;{\hat{\Sigma }}_{{}_{t-1}}=\;\partial_{\vec{h}}\vec{g}\left({\vec{\mu }}_{t-1}\right){\text{ Σ}}_{t-1}\partial_{\vec{h}}{\vec{g}}^{T}\left({\vec{\mu }}_{t-1}\right)+Q,$$

$$Y_{t}=\left[{⊕}_{i=1}^{6}\pi_{i}\left({\vec{g}}^{\left(e\right)}\left({\vec{\mu }}_{t-1}\right)\right)-\vec{\pi }\left({\vec{g}}^{\left(e\right)}\left({\vec{\mu }}_{t-1}\right)\right)\vec{\pi }{\left({\vec{g}}^{\left(e\right)}\left({\vec{\mu }}_{t-1}\right)\right)}^{T}\right]⊕0_{3,3},$$ (12)

where 0_3,3_ denotes squared null matrix and $\partial_{\vec{h}}\vec{g}({\vec{\mu }}_{t-1})$ denotes the Jacobian matrix of $\vec{g}\left(\vec{h}\right)$ computed at prior expectations ${\vec{\mu }}_{t-1}.$

There are two interesting features of these update equations:

- The update equation for the posterior expectation Eq (11) have the form of a WTA neural network, with the key feature that the external input is proportional to the prediction error. This is similar to the hierarchical neuronal network models used in [15,25] to model behavioral planning in prefrontal cortex. The important difference is that in our model the update equations are derived from a probabilistic generative model (see Eq (8)), and therefore there is an adaptive influence of prediction errors on the internal dynamics of the WTA network; as expected from the Bayesian observer assumption.
- The hypothesis evidence $p\left({\vec{e}}_{t}|H_{t}\right)$ is modulated by the predicted relevance of that hypothesis ${\vec{g}}^{\left(e\right)}\left({\vec{\mu }}_{t-1}\right)$ when the posterior hypothesis probability ${\vec{\rho }}_{t}$ is computed (see Eq (9)). Effectively, the evidence in favor of a hypothesis is neglected if the expectation about its relevance is low. This is similar to the effect that attention has on the processing of sensory information, as only the currently relevant features of the stimuli are being processed at any moment of time. Importantly, in the presence of competitive inhibitory dynamics the expectations of all but the most likely hypothesis will be suppressed. In other words, internal dynamics of beliefs leads to selection of prior expectation [34].

As the derivation of the perceptual model required multiple assumptions, which are not directly motivated by the behavioral data, it is important to test which of the assumption is actually essential for describing and predicting behavioral responses. Thus, in what follows we will describe several variants of the perceptual model that are obtained by relaxing some of the assumption made in the derivations presented above.

#### Structured models

To reduce the number of free parameters in the perceptual model described above we will assume that between the 2^nd^ and the 3^rd^ level there are only symmetric excitatory connections with equal values and that these connections exist only between components encoding the relevance of exemplars and corresponding visual features, thus

$${\left[W_{dist}^{\left(f\right)}\right]}_{i,j}={\left[W_{dist}^{\left(e\right)}\right]}_{j,i}=\{\begin{array}{c} w_{dist},\text{ for }v\left(i\right)=j\\ 0,\text{ for }v(i)\neq j \end{array},$$

where *v*(*i*) maps the *i* th exemplar to the corresponding visual feature. Furthermore, we will assume that within the 2^nd^ and the 3^rd^ level there are only symmetric inhibitory connections with equal values, thus

$${\left[W_{lat}^{\left(e,f\right)}\right]}_{i,j}=\{\begin{array}{c} -w_{lat}^{\left(e,f\right)},\text{ for }i\neq j\\ 0,\text{ for }i=j \end{array}.$$

Importantly, we will constrain the WTA dynamics to attractor states in which only single component of $\;{\vec{h}}_{t}^{\left(e\right)}$ and ${\vec{h}}_{t}^{\left(f\right)}$ have high values while all other components are set to zero or lower values. This is achieved by setting $w_{lat}^{\left(e,f\right)}=2\kappa_{e,f}$, as suggested in [41].

However, removing the lateral inhibition form either the 2^nd^ or the 3^rd^ level would not disrupt completely the attractor dynamics as long as there are excitatory connections between levels. Thus, we will also consider two additional variants of the structured model in which we set either *κ*_*e*_ or *κ*_*f*_ to zero.

**Fig 5:**
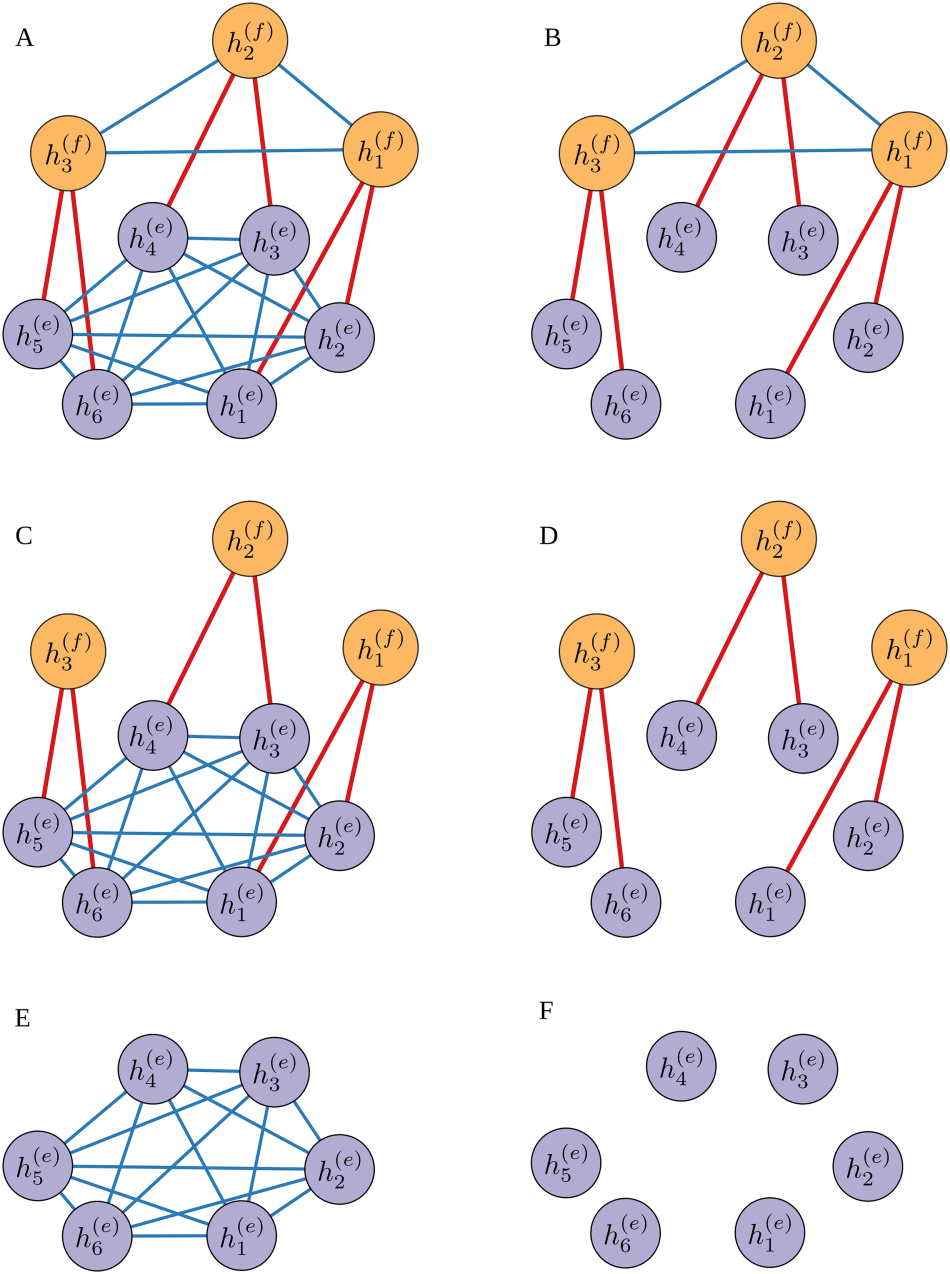
Visualization of six different model structures. Graphical representations of the connectivity matrix *W* of all variants of the perceptual model: the three variants of the structured model denoted with (A) *w*_1_, (B) *w*_2_, and (C) *w*_3_; (D) the structure-free model variant denoted with *d*; and the two reduced variants of the perceptual model denoted with (E) *rw*, and (F) *rd* (for formal definition please see the accompanying text). The relevance of visual features and exemplars encoded by the vector $\;{\vec{h}}_{t}=\left({\vec{h}}_{t}^{\left(e\right)},{\vec{h}}_{t}^{\left(f\right)}\right)$ (see Fig 4) corresponds to the activity levels at the nine nodes of the neural network. The orange nodes encode the relevance of three visual features ${\vec{h}}_{t}^{\left(f\right)}$(color, motion, and shape). The purple nodes encode the relevance of six exemplar-feature pairs ${\vec{h}}_{t}^{\left(e\right)}$ (red-color, green-color, leftward-motion, rightward-motion, circle-shape and square-shape). The structured models incorporate symmetrical lateral inhibition $\;w_{lat}^{\left(e,f\right)}$ (depicted with blue lines) that implements a winner-take-all dynamics (see Eq (6) and Eq (7)) and symmetrical excitation between levels *w*_*dist*_ (depicted with red lines), that implement integration of relevance between levels of hierarchy. Note that the structure-free model has only symmetrical excitation *w*_*dist*_ (red lines) from the level of exemplar-feature pairs to the level of visual features. In the case of the reduced perceptual models, the level of visual features is removed.

Therefore, the full set of parameters of the structured perceptual models is given by $\gamma =\left\{\varepsilon ,\tau_{e,f},\kappa_{e,f},q_{e,f},w^{dist},{\vec{\mu }}_{e,f}^{0},\sigma_{e,f}^{0}\right\}$, where in the first variant, denoted by *w*_1_, we have that *κ*_*e*,*f*_ ≠ 0, in the second variant, *w*_2_, we set *κ*_*e*_ = 0, and in the third variant, *w*_3_, we set *κ*_*f*_ = 0. The graphical representation of all structured model variants is shown in Fig 5A–5C.

#### Structure-free model

To explicitly test whether a complex attractor dynamics is necessary to describe subjects’ behavior, that is, to test whether an attention-like mechanism modulates the update of beliefs, we require an alternative model without such an attentional focus mechanism. Hence, by setting both *κ*_*e*_ and *κ*_*f*_ to zero we obtain a structure-free model, denoted by *d*, in which the state transition of $\;{\vec{h}}_{t}$ is described with a diffusive dynamics (Fig 5D). The effect of removing the lateral inhibition is that a feature considered relevant will not inhibit other features, that is, there is no attentional focus effect. Note that setting *κ*_*e*,*f*_ = 0 also reduces the number of free parameters, thus the model complexity. Critically, by employing a model with lower complexity enables us to test whether the attentional focus model may be too complex for the behavioral data.

Note that both the structured and the structure-free models are able to capture the transient relevance of visual features. However, one expected difference is that the structured model, as it encodes a key constraint of the task environment, requires less evidence to form strong beliefs about relevance of visual features.

#### Reduced structured and structure-free models

To further simplify both structured and structure-free models note that the 3^rd^ level of the hierarchy encodes the beliefs about the relevance of a visual feature. The importance of the 3^rd^ level is to provide, as a dynamical implementation, the integration of the beliefs from the 2^nd^ level of the hierarchy. The expectations at the 3^rd^ level of the hierarchy are then used to generate responses, as described in the text below. In addition, one can also generate responses by using directly the expectations provided at the 2^nd^ level of the hierarchy. In such a case the 3^rd^ level of hierarchy is obsolete and can be removed.

In this way we obtain two reduced variants of the perceptual model defined by the following set of the free parameters $\left\{\varepsilon ,\tau_{e},\kappa_{e},q_{e},{\vec{\mu }}_{e}^{0},\sigma_{e}^{0}\right\}$. For the reduced structured model, denoted by *rw*, *κ*_*e*_ is a free parameter (Fig 5E), while for the reduced structure-free model, denoted by *rd*, *κ*_*e*_ is fixed to zero (Fig 5F).

Non-Bayesian perceptual models. All the previous variants of the perceptual model were based on the same form of the update equations as provided in Eqs (11) and (12). The only difference so far between them is that certain parameters were removed, that is, fixed to zero. Importantly, these update equations are based on the assumption that subjects combine prior beliefs and sensory information in a Bayes optimal fashion. This requires the representation of both the expectations about the true state of the world and the uncertainties about these expectations. This assumption might not be correct in our case, and potentially the only relevant quantity, both for update of beliefs and for generating responses, might be the expectations about the relevance of exemplars and visual features. Thus, to test for this possibility we considered a non-Bayesian variant of the perceptual model described above, in which we fix the values of prior and posterior uncertainty on all levels of the hierarchy. This effectively makes the perceptual model non-Bayesian, as the sensory observations are not combined with the prior knowledge in a Bayes-optimal fashion. Thus, in the non-Bayesian variant of the perceptual model, we will set the posterior covariance matrix to a fixed value, Σ_*t*_ = *αI*_9_, which leads to the following update equations of expectations

$$\begin{array}{c} {\vec{\mu }}_{t}^{\left(e\right)}={\vec{g}}^{\left(e\right)}\left({\vec{\mu }}_{t-1}\right)+\alpha \left({\vec{\rho }}_{t}-\vec{\pi }\left({\vec{g}}^{\left(e\right)}\left({\vec{\mu }}_{t-1}\right)\right)\right),\\ {\vec{\mu }}_{t}^{\left(f\right)}={\vec{g}}^{\left(f\right)}\left({\vec{\mu }}_{t-1}\right). \end{array}$$ (13)

Furthermore, in this formulation the evidence *ρ*_*t*,*k*_ = 1 − *ϵ* if the exemplar supporting *k*th hypothesis was selected and *ρ*_*t*,*k*_ = *ϵ* otherwise, where $\epsilon \in \left[0,\;\frac{1}{2}\right]$ denotes a free parameter which is not equivalent to the experimenters error rate *ε*, but only related to it. Note that the update equations shown in Eq (13) have a functional form similar to the Rescorla-Wagner model which is often used in reinforcement learning models [68,69].

#### Response model

Having obtained the update equation for the hidden belief states, the next step is to define an appropriate response model (see Fig 3). Thus, the question we will answer here is what would be an optimal response in an experimental trial *t* given the hidden beliefs *b*_*t*_? Note first that the posterior probability that the *i*th visual feature is currently relevant is defined as

$$p_{t,i}=\frac{e^{\mu_{t,i}^{\left(f\right)}}}{\sum_{j=1}^{3}e^{\mu_{t,j}^{\left(f\right)}}},$$

in the case of the perceptual model variants with the 3^rd^ level of hierarchy, and

$$p_{t,i}=\frac{e^{\mu_{t,i_{1}}^{\left(e\right)}}+\;e^{\mu_{t,i_{2}}^{\left(e\right)}}}{\sum_{j=1}^{6}e^{\mu_{t,j}^{\left(e\right)}}},$$

in the case of the reduced perceptual model variants without the 3^rd^ level (where *i*_1_ and *i*_2_ denote the positions of the exemplars of the corresponding *i*th visual feature).

Importantly, as described above, we have instructed the subjects that at the end of the experiment one of the experimental trials will be randomly selected and the subject will receive as a reward the money that they have assigned to the truly relevant visual feature. Thus, we will assume that the subject’s responses depend on the subject’s risk attitude. As various studies have demonstrated that humans exhibit variable risk tendencies [70–73], we will parametrize the subject’s individual levels of risk aversion with an inverse risk factor *θ*_1_. Using the formalism of the Bayesian decision theory (BDT) and under the assumption that a subject’s absolute risk aversion is inversely related to the outcome of the bet, we have derived theoretical evidence that the optimal response (for more details see S2 Text) is defined as

$${\vec{r}}_{t}=\frac{{\vec{p}}_{t}{}^{\theta_{1}}}{\sum_{j=1}^{3}p_{t,j}{}^{\theta_{1}}},$$ (14)

where the elements of the response vector ${\vec{r}}_{t}$ denote the fraction of money assigned to the corresponding visual feature. Note that the higher the *θ*_1_ is the more money will be assigned to the visual feature with highest posterior probability *p*_*t*,*j*_, hence the higher the *θ*_1_ the riskier is the subject’s behavior. In the limit of *θ*_1_→0, the responses become independent of the posterior beliefs and the same amount of money is always assigned to all visual features, thus reflecting infinite risk aversion.

However, using the optimal response function to model subjects’ behavior may be too restrictive, as the behavioral responses might deviate from the optimal responses for at least two reasons: First, the perceptual models proposed might not fully capture the hidden perceptual processes of human subject, thus there might be an unknown influences on the decision process. Second, recent findings suggest that human brain maintains only stochastic representation of posterior beliefs [51]. In other words, an exact representation of posterior expectations is not internally available to the subject. Thus, under an assumption that the posterior expectations are sampled stochastically, one expects that the deviation of the response from the optimal one is proportional to the posterior uncertainty [51].

To account for potential deviation from optimal response we will define the behavioral responses as

$${\vec{r}}_{t}=\frac{{\vec{p}}_{t}{}^{\theta_{1}}e^{{\vec{\xi }}_{t}}}{\sum_{j=1}^{3}p_{t,j}{}^{\theta_{1}}e^{\xi_{t,j}}},$$ (15)

where ${\vec{\xi }}_{t}$ denotes a vector of i.i.d. random variables representing perturbations to the optimal response. We will assume here that the perturbation term $\vec{\xi }$ has two components expressed as separate components of the covariance matrix of a zero-mean Gaussian distribution:

$${\vec{\xi }}_{t}\sim N\left(0,P_{t}\right);\;P_{t}=\theta_{2}I_{3}+\theta_{3}\Sigma_{t}^{\left(f\right)}.$$ (16)

The first noise source represents unknown influences on the decision process, which we assume to be i.i.d. The second noise source, which represents the above stochastic sampling assumption, is proportional to the uncertainty about the expected relevance of the visual features. Note that the second component is only relevant for the probabilistic variants of the perceptual model with full hierarchical representation, as only in those cases is the posterior uncertainty about the feature relevance a dynamic quantity. Consequently, the full set of the parameters for the response model *m* ^(*r*)^ becomes *θ* = {*θ*_1_,*θ*_2_,*θ*_3_}.

Finally, for the above defined response model the response likelihood is defined as the multivariate logistic-normal distribution, that is,

$$p\left({\vec{r}}_{t}|b_{t}(\gamma ),\theta \right)=\frac{1}{3r_{t,1}r_{t,2}r_{t,3}\cdot Z\left({\vec{m}}_{t},\;P_{t}\right)}N\left(clr\left({\vec{r}}_{t}\right);{\vec{m}}_{t},P_{t}\right).$$

Here $clr\left({\vec{r}}_{t}\right)$ denotes the centered log-ratio transform

$$clr\left({\vec{r}}_{t}\right)=\text{ln}\left(\frac{{\vec{r}}_{t}}{\sqrt[{3}]{\prod_{i=1}^{3}r_{t,i}}}\right),$$

$Z\left({\vec{m}}_{t},P_{t}\right)$ denotes a normalization constant, and ${\vec{m}}_{t}=\theta_{1}{\vec{\mu }}_{t}^{\left(f\right)}$ in the case of the full perceptual model or ${\vec{m}}_{t}=\theta_{1}clr\left({\vec{p}}_{t}\right)$ in the case of the reduced perceptual model.

The normalization constant is computed as

$$Z\left({\vec{m}}_{t},P_{t}\right)=\underset{\mathbb{R}}{∭}\delta ({\vec{a}}^{T}\cdot {\vec{x}}_{t})N\left({\vec{x}}_{t};{\vec{m}}_{t},\;P_{t}\right)d{\vec{x}}_{t}=\frac{1}{\sqrt{2\pi \left({\vec{a}}^{T}P_{t}\vec{a}\right)}}\text{exp}\left(-\frac{{\left({\vec{a}}^{T}{\vec{m}}_{t}\right)}^{2}}{2\left({\vec{a}}^{T}P_{t}\vec{a}\right)}\right),$$

where the projection vector $\vec{a}={\left(1,1,1\right)}^{T}$. The normalization constant is required because of the mapping of the space of posterior expectations ${\vec{\mu }}_{t}^{\left(f\right)}\in {\mathbb{R}}^{3}$ onto a 2D simplex, which is the space of responses $\Delta^{2}=\left\{{\vec{r}}_{t}\in {\mathbb{R}}^{3}|\overset{3}{\underset{i=1}{\sum }}r_{t,i}=1,\;r_{t,i}\geq 0\text{ for }\forall \;i\right\}\;$.

For model comparisons, we will consider two response models. For both models, all the equations in this section apply, but the critical difference is that we only allow *θ*_3_ as a free parameter in the so-called full response model, while in the reduced response model we fix *θ*_3_ at 0. The effect of this difference is that the reduced model assumes a constant response variability of subjects, while the full response model allows for response variability to be dependent on the internal uncertainty about feature relevance. Note that having the inverse risk factor *θ*_1_ as a free parameter in all variants of the response model is a result of a preliminary analysis (not presented here) which showed that response model variants with fixed risk factor have substantially lower model evidence compared to the considered variants of the response model.

### List of models and model evidence computation

For the model comparison, we have paired all the full variants of the Bayesian perceptual models with the two variants of the response model; the reduced variants of the Bayesian models and all the variants of the non-Bayesian perceptual models were paired only with the reduced response model, as the posterior uncertainty about the visual features $\Sigma_{t}^{\left(f\right)}$ is set to constant values in this cases. In addition, we have defined a simple baseline model. Hence in total we consider 17 behavioral models denoted as:

1. *BM*—Baseline model in which the beliefs and the uncertainties about the beliefs are assumed to be constant over time. Thus, all the parameters of the perceptual model are set to zero, except ${\vec{\mu }}_{f}^{0}$. Similarly, we fixed *θ*_1_ = *θ*_2_ = 1 as they are redundant for this case and leave only *θ*_3_ as the free parameters of the response model. The role of the baseline model here is to provide for a trivial explanation to the behavioral data: subjects generated random responses around a fixed mean independent from the sensory cues.
2. $B_{rw,rd,\;d,\;w_{1},\;w_{2},w_{3}}^{f,\;r}$—Twelve different Bayesian perceptual models, where the superscript denotes the variant of the response model (*f*→*θ*_2_> 0, *r*→*θ*_2_ = 0), and the subscript denotes the variants of the perceptual model (*rw*→ reduced perceptual model with lateral inhibition, *rd*→ reduced perceptual model without lateral inhibition, *d*→ full perceptual model without inhibition at all levels, *w*_1_→ full model with lateral inhibition on all levels, *w*_2_→ full model with lateral inhibition only at the 2^nd^ level, *w*_3_→ full model with lateral inhibition only at the 3^rd^ level), see Fig 5.
3. $NB_{rw,rd,\;d,\;w_{1},\;w_{2},w_{3}}^{\;r}$—Six different non-Bayesian perceptual models, where the superscript denotes the only possible variant of the response model, the reduced response model, and the subscripts denote the variants of the perceptual model, with the same notation as above.

To summarize the motivation for these different variants of the perceptual model (see Methods above for details): the structure-free model variants test for the possibility that the structured representation is not required for describing the behavioral data; the model variants without the final level of the hierarchy (*rw*,*rd*) test for the possibility that the final level of hierarchy is redundant for describing the behavior; the non-Bayesian variants of the perceptual test for the possibility that the Bayesian observer assumption is not required for describing the behavior.

Each model variant is defined using a set of free parameters {*γ*,*θ*} for the perceptual and response models. To be able to define prior and posterior distributions in the same functional form of multivariate normal distributions, we transform all parameters so that they have the same domain of real numbers. Note that such a transformation does not change the value of model evidences, as to compute the model evidence one integrates over all the free parameters of a generative model. Let us denote by $\vec{\chi }$ the vector of perceptual and response parameters transformed to real space, then $\vec{\chi }=\left(\vartheta \left(\gamma \right),\vartheta \left(\theta \right)\right)$, where

$$\vartheta \left(z\right)=\{\begin{array}{c} \text{ln}\left(z\right),\;\;if\;z\;\in \left\{\alpha ,\kappa_{e,f},q_{e,f},w^{dist},\sigma_{e,f}^{0},\theta_{1},\theta_{2},\;\theta_{3}\right\}\\ \text{ln}\left(\frac{2z}{1-2z}\right),\;\;if\;z=\varepsilon \;\\ \text{ln}\left(\frac{2z-1}{2(z-1)}\right),\;\;if\;z=\tau_{e,f}\;\\ z,\;\;\;if\;z\in \left\{{\vec{\mu }}_{e}^{0},{\vec{\mu }}_{f}^{0}\right\} \end{array}.$$

Thus, we can define the prior distribution over model parameters as a multivariate normal distribution $N\left(\vec{\chi };{\vec{\eta }}_{0},s_{o}I\right)$.

The log-joint probability distribution can then be written as

$$l\left(\vec{\chi }\right)=\overset{T}{\underset{k=1}{\sum }}\text{ln}p\left({\vec{r}}_{k}|b_{k}\left({\vec{e}}_{k},\vartheta^{-1}\left({\vec{\chi }}_{\gamma }\right)\right),\vartheta^{-1}\left({\vec{\chi }}_{\theta }\right)\right)+\text{ln}N\left(\vec{\chi };{\vec{\eta }}_{0},s_{o}I\right),$$ (17)

where *T* denotes the number of trials within a single experimental block. The Laplace approximation to the log-evidence is obtained as

$$\text{ln}p\left(r_{1\ldots t}|{\vec{e}}_{1\ldots t}\right)=l\left(\vec{\beta }\right)+\frac{1}{2}\text{ln}\left|2\pi S\right|,$$ (18)

where $\vec{\beta }$ denotes the mode of $\;l\left(\vec{\chi }\right)$ and $S=-\partial_{\vec{\chi },\vec{\chi }}l{\left(\vec{\chi }\right)}^{-1}{|}_{\vec{\chi }=\vec{\beta }}$, *i*.*e*. S is the negative inverse of the Hessian matrix at the mode $\vec{\beta }$.

To find the mode of $l\left(\vec{\chi }\right)$ we applied the so-called Covariance Matrix Adaptation Evolution Strategy (CMA-ES). CMA-ES is a numerical optimization method, which has been applied successfully in various research areas [74–77] and is particularly useful for ill-conditioned and multimodal objective functions. In short, CMA-ES is a stochastic derivative-free method for numerical optimization of non-linear optimization problems [56,57]. We used a freely available Matlab toolbox that implements the algorithm [Hansen, Nikolaus (2004). (https://www.lri.fr/~hansen/cmaes_inmatlab.html#matlab), Version 3.61].

Once the mode of the log-joint probability distribution (Eq (17)) is found, we have to estimate the curvature at the mode, that is, the Hessian matrix. We estimated the Hessian matrix by numerical differentiation [58], where we used the following toolbox [D’Errico, John (2006). (http://www.mathworks.de/matlabcentral/fileexchange/13490), MATLAB Central File Exchange. Retrieved 10. November 2013].

Because of the stochastic nature of the CMA-ES algorithm we repeated the stochastic search *N* = 50 times per experimental block for each model. For each of the *N* solutions we estimated the Hessian matrix and computed the Laplace approximation to the log-evidence. Finally, we kept the solution with the largest log-evidence, therefore increasing the probability of finding the maximal lower bound to the log-evidence and thus the most likely model of a subject’s behavior. The numerically obtained $\vec{\beta }$ and *S* are used as the mean and the covariance matrix of the approximate posterior distribution $N\left(\vec{\chi };\vec{\beta },S\right)$. Note that in this way we obtain the full covariance matrix without the need for a mean field approximation, which would neglect any existing correlations between parameters. All data processing was performed using MATLAB [version 8.1, The MathWorks Inc., Natick, Massachusetts].

### Bayesian model selection

We first estimated the log model evidence of the 17 generative models described above for each experimental block. To obtain a total per-subject log-evidence for each experimental condition, we summed the estimated log-evidences over experimental blocks of a single experimental condition. This gives us the log model evidence of each generative model for each subject per experimental condition. We used the obtained log-evidences to apply the hierarchical Bayesian model selection approach described in [54,55]. By using hierarchical Bayesian model selection we assumed that the identity of the best-fitting model may vary across subjects. This requires treating the posterior model probability (the posterior belief that a given model has generated the data) as a random variable.

Thus, the two computed quantities of interest are the expected probability (EP) and the exceedance probability (XP) of each model: The EP is defined as the probability that a given model generated the behavioral data of a randomly selected subject (see [55] for a detailed mathematical description); The exceedance probability XP tells how likely it is that a given model will have the largest probability in a random sample from the posterior distribution. Importantly, the XP can be seen as a degree of confidence in the difference between posterior model probabilities [55]. Thus, when presenting the results of a model comparison we will only report the XP of the corresponding model or model family, as large XP at the same time implies significantly larger EP. Importantly, we will only consider recently proposed “protected” exceedance probability, which takes into account the null hypothesis that assumes that all the models are equally likely (see [55] for details). We will consider that the EP of a single generative model is significantly larger than the EP of other generative models, if the model’s XP is above threshold value set at 0.95. Although, this threshold value was selected in the analogy to classical statistical tests that rely on p-values, its relation to the statistical power is not equivalent (see [55]).

We used the MATLAB implementation of the random-effect Bayesian model selection [(https://sites.google.com/site/jeandaunizeauswebsite/code/rfx-bms), retrieved January 2014]. In what follows we will describe the results obtained by applying the Bayesian model selection to the set of behavioral models that we used to approximate subjects’ behavior in the probabilistic WCST.

## Results

**Fig 6:**
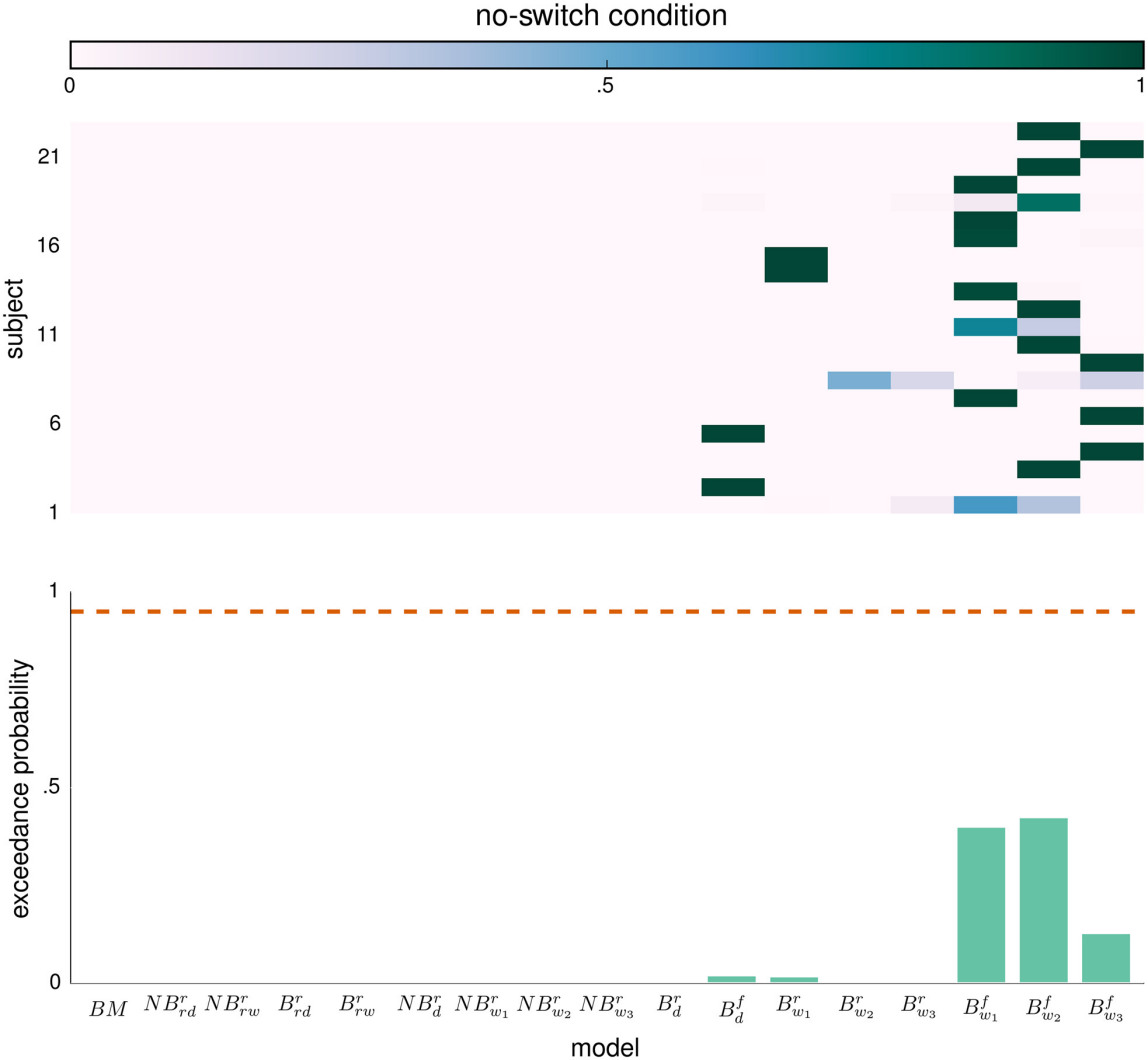
Random-effects model comparison for the no-switch condition. (top) Posterior model probability (see color bar) for each subject. For an exact description of each of the 17 models see main text. (bottom) Exceedance probability (XP) that a given model is more likely to generate the data than any other model. The dashed orange line denotes the confidence threshold level set at 0.95.

**Fig 7:**
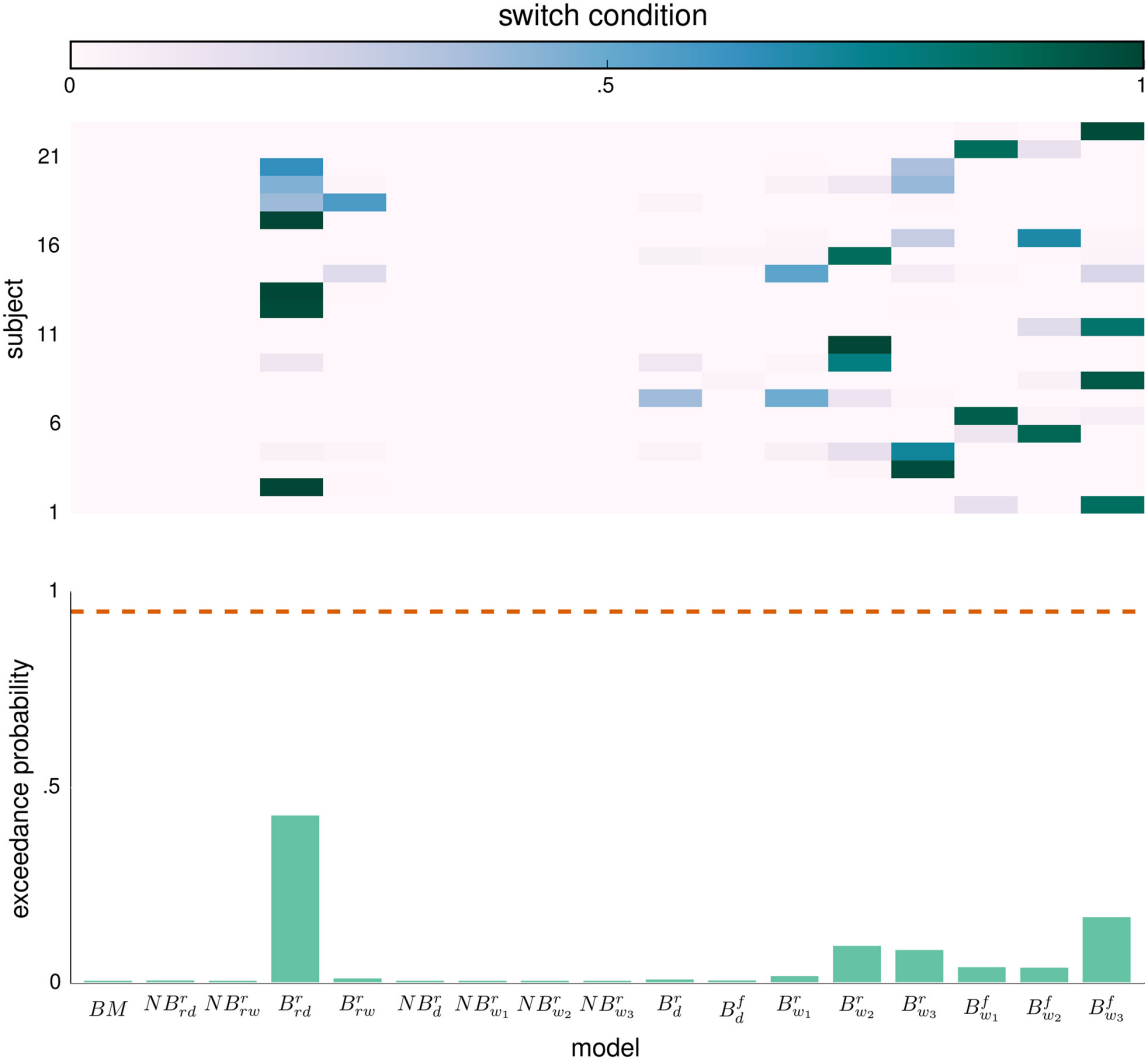
Random-effects model comparison for the switch condition. (top) Posterior model probability (see color bar) for each subject. For the exact description of each of the 17 models see main text. (bottom) Exceedance probability (XP) that a given model is more likely to generate the data than any other model. The dashed orange line denotes the confidence threshold level set at 0.95.

In Figs 6 and 7 we present the results of the random-effects Bayesian model comparison at the group-level. We have separated the model comparison between the two experimental conditions, switch and no-switch. We estimated the per-subject log-evidence for each experimental condition as the sum of log-evidences across the three corresponding experimental blocks. The top graph in both Figs 6 and 7 depicts the model attributions to the behavioral responses of each subject, that is, the posterior probability that a given model has generated the behavioral responses of each subject, for each condition separately. The bottom graphs show the corresponding XP for each of the 17 models. The direct comparison of behavioral models is inconclusive, as the highest XP is in both cases below the threshold value. Note that this is a typical issue when the model comparison set contains groups of closely related models [78].

The solution here is that instead of trying to answer which of the models provides the best description of behavioral data, we should ask which of the features of the perceptual and the response model are the most relevant for generating the data [78]. Note that in both figures we observe clustering of high model probabilities (top graphs) within closely related perceptual models (e.g. $B_{\;w_{1},w_{2},w_{3}}^{f}$) which only differ in the type of the connectivity matrix (see subsection Structured models in Methods). Thus, to determine which of the features of the perceptual and the response model are the most relevant for generating the behavioral data, we have performed four so-called family-wise model comparisons [78]. To test whether non-Bayesian or Bayesian model variants better describe the behavioral data, we grouped all models into baseline (BM = {*BM*}), non-Bayesian $\left(\text{NB}=\left\{NB_{rw,rd,d,w_{1},w_{2},w_{3}}^{r}\right\}\right)$ and Bayesian $\left(\text{B}=\left\{B_{rw,rd,d,w_{1},w_{2},w_{3}}^{f,r}\right\}\right)$ model families. Similarly, to test whether a hierarchical representation of feature relevance is truly necessary we have grouped the models into BM, reduced perceptual $\left(\text{RP}=\left\{NB_{rw,rd}^{r},B_{rw,rd}^{r}\right\}\right)$, and full perceptual $\left(\text{FP}=\left\{NB_{d,w_{1},w_{2},w_{3}}^{r},B_{d,w_{1},w_{2},w_{3}}^{f,r}\right\}\right)$ model families. Finally, to test whether the attractor dynamics contributes to an explanation of the behavioral data, we have grouped models into the BM, structure-free $\left(\text{SFM}=\left\{NB_{rd,d}^{r},B_{rd}^{r},B_{d}^{f,r}\right\}\right)$, and structured $\left(\text{SM}=\left\{NB_{rw,w_{1},w_{2},w_{3}}^{r},B_{rw}^{r},B_{w_{1},w_{2},w_{3}}^{f,r}\right\}\right)$ model families. In addition to separating behavioral models based on the features of perceptual model, we have grouped them based on the features of the response model, for which we considered only two model families, a model family with the reduced response model $\left(\text{RR}=\left\{BM,NB_{rw,rd,d,w_{1},w_{2},w_{3}}^{r},B_{rw,rd,d,w_{1},w_{2},w_{3}}^{r}\right\}\right)$ and a family with the full response model $\left(\text{FR}=\left\{B_{rw,rd,d,w_{1},w_{2},w_{3}}^{f}\right\}\right)$.

**Fig 8:**
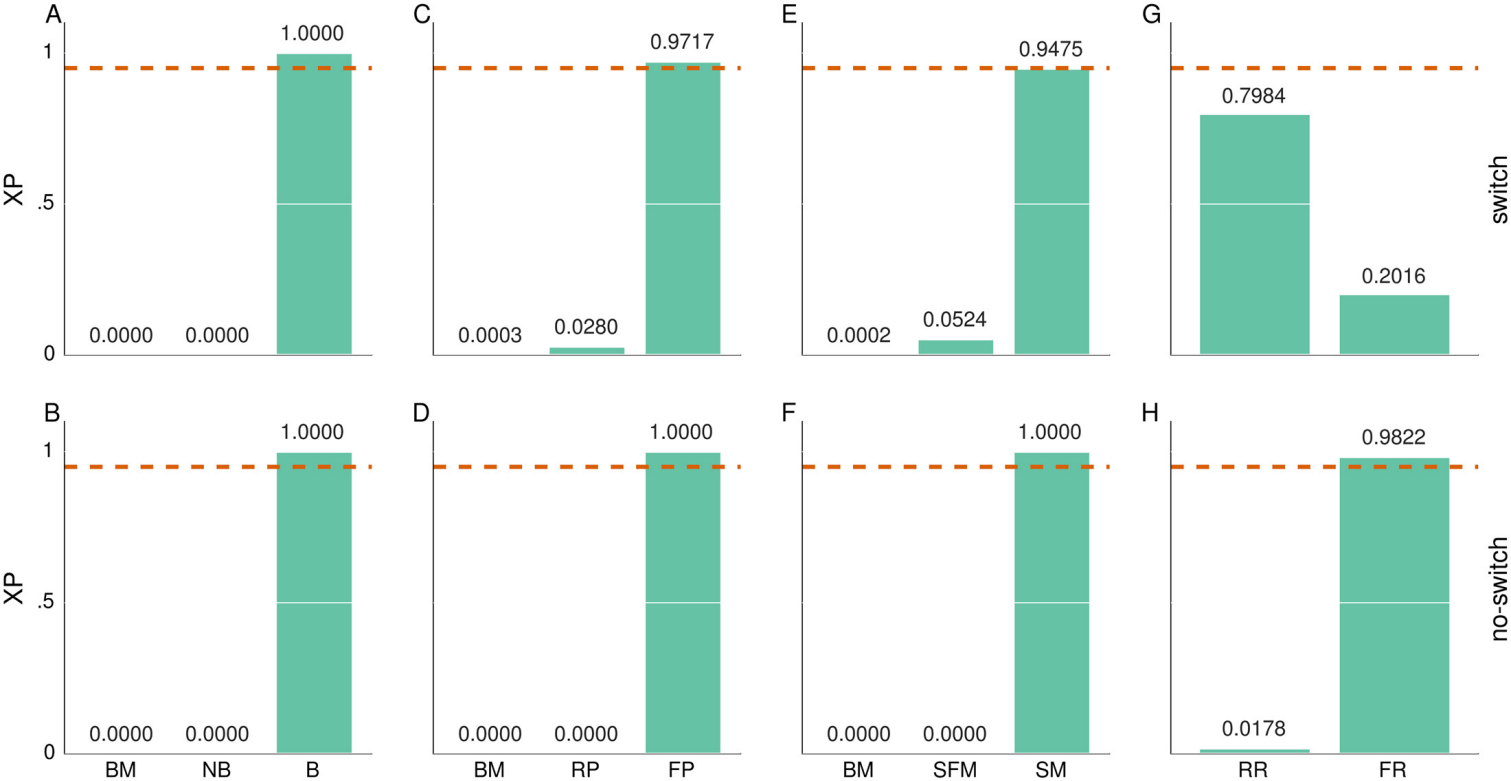
Family-wise model comparisons. (A-B) Exceedance probability (XP) of the baseline model (BM) non-Bayesian (NB) and Bayesian (B) model families. (C-D) XP of the BM, reduced perceptual (RP) and full perceptual (FP) model families. (E-F) XP of the BM, structure-free (SFM) and structured (SM) model families. (G-H) XP of the reduced response (RR) and full response (RR) model families. The top graphs (A,C,E,G) show the exceedance probability of model families for the switch condition, whereas the bottom graphs (B, D, F, H) show the exceedance probability of the model families for the no-switch condition. The dashed orange lines denote the confidence threshold level set at 0.95.

**Fig 9:**
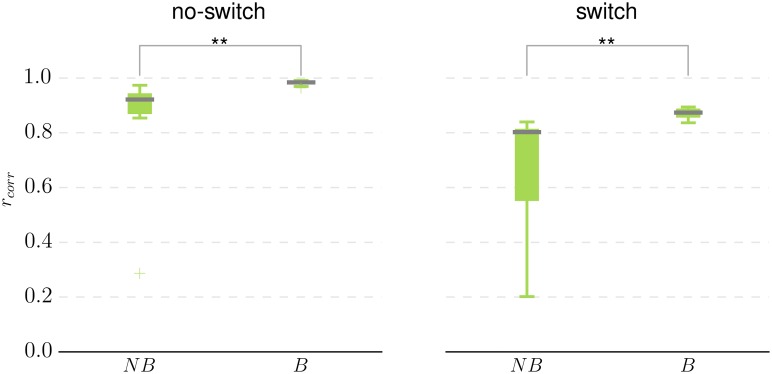
Distribution of the correlations between the mean model performance and the mean subjects’ performance across two model families. Boxplot of the Pearson correlation coefficient *r*_*corr*_ estimated for each model within the non-Bayesian (NB) and the Bayesian (B) model families in the no-switch and switch condition. For each model within each family we have computed the Pearson correlation coefficient between the mean model performance and mean subjects’ performance. In both conditions the non-Bayesian model family has a significantly lower median correlation (denoted by a dark horizontal line within the boxes) with *p*<0.005 (Kruskal-Wallis test).

From the results of the four family-wise model comparisons, shown in Fig 8, we can conclude with high confidence (XP above the threshold level of 0.95) that the Bayesian formulation of the perceptual model is essential for generating behavioral data in both experimental conditions (see Fig 8A and 8B). To understand the difference between NB and B model families in their ability to predict subjects’ behavior we tested how well the behavioral models within each of these families predict subjects’ performance. We computed the mean model performance by first estimating the expected performance per trial. To do this, we fixed model parameters to the mode $\vec{\beta }$ of the posterior parameter distribution and computed the expected model response; hence the expected performance per trial corresponds to the mean fraction of money assigned to the truly relevant visual feature at that trial. We averaged the per-trial expected model performance over a whole experimental block to obtain the mean model performance per experimental block. We then estimated the Pearson correlation coefficient between the mean model performance and mean subjects’ performance across blocks and both experimental conditions. In Fig 9 we illustrate, with a box plot, the distribution of the estimated correlation within NB and B model families. The correlation coefficient shows that, on average, the NB model family has significantly lower correlation with subjects’ performance, or in other words, the NB model family provides a worse fit to subjects’ behavior compared to the Bayesian model family. Interestingly, within the NB family the models with consistently low correlation, in both conditions, are the structure-free model variants $NB_{d}^{r}$ and $NB_{rd}^{r}$ (see S1 Fig), whose update equation correspond to what is typically used in classical reinforcement learning models. On the other hand, the non-Bayesian model variants with attractor dynamics, namely $NB_{rw,w_{1},w_{2},w_{3}}^{r}$, show consistently high correlation with subjects’ performance in both conditions (with one exception being model $NB_{w_{2}}^{r}$). This indicates that even only within the NB model family the attentional focus mechanism plays a critical role in replicating subjects’ behavior.

Importantly, from the results of the family-wise model comparison we can also conclude with high confidence that the full variant of the perceptual model (including both the 2^nd^ and 3^rd^ level of the hierarchy, see Reduced structured and structure-free models for details) is an essential feature in both experimental conditions (see Fig 8C and 8D). The structured family of the perceptual model shows an XP above the threshold level only in the no-switch condition (Fig 8F), whereas in the switch condition the XP is slightly below the confidence threshold level (Fig 8E), but still high enough to be considered a trend. One possible explanation for the slightly reduced confidence in the structured model family (Fig 8E) is that in the switch condition one expects high levels of posterior uncertainty about the relevance of visual features. This is due to an increased difficulty in assigning contradicting evidence either to an experimenter’s error or a change in the selection rule. Thus, in such an environment one does not expect that a subject can form strong beliefs about the relevance of each visual feature. Hence the attractor dynamics would not show strong advantages in generating the data, when compared to the structure-free model family.

Finally, when comparing model families with the full against the reduced variant of the response model we get mixed results across conditions. The full response model seems to be relevant for generating behavioral data only in the no-switch condition (Fig 8H), whereas in the switch condition the evidence is inconclusive (Fig 8G). This discrepancy between the confidence levels in the two experimental conditions may be caused by the increased difficulty of the switch task, which effectively introduced a higher variability in subjects’ responses. Most of this variability may be explained simply by a high but constant level of response noise as formulated in the reduced response model.

**Fig 10:**
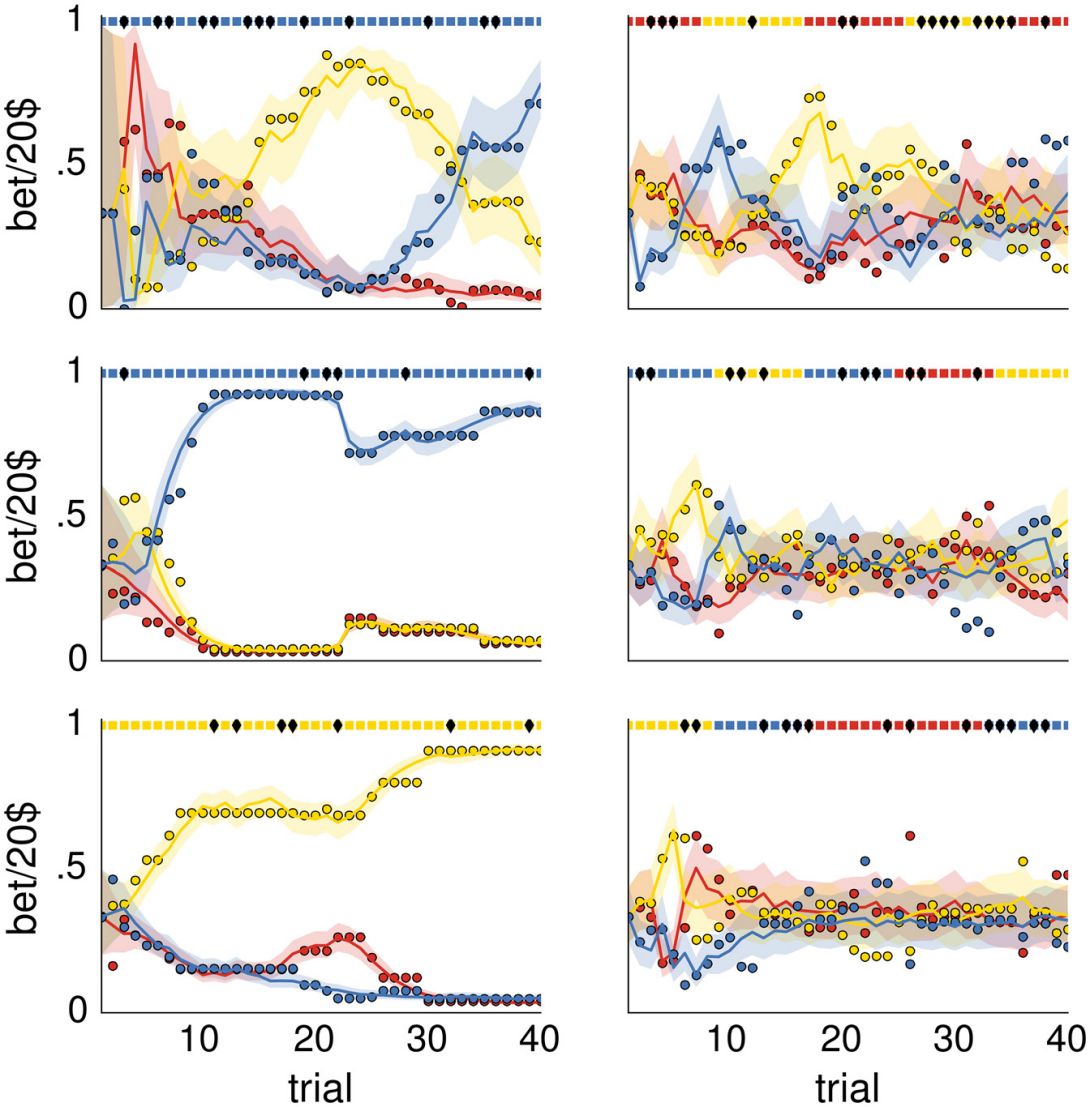
Behavioral responses and modeled responses for a representative single subject. (left) The three no-switch blocks, and (right) the three switch blocks. Colored circles denote the behavioral responses of subject #9 obtained as the fraction of money assigned to each of the three visual features on single trials. Solid lines denote the expected model response computed at the mode $\vec{\beta }$ of the posterior distribution over model parameters and averaged over posterior model probabilities for subject #9 (see Figs 6 and 7). The shaded area corresponds to the 95% probability interval. Each color corresponds to one of the three visual features (red—color, yellow—motion, blue—shape). The dotted colored line at the top of each plot denotes the relevant visual feature during each experimental trial, where the black diamond marks on the dotted line denote trials in which the experimenter selected the wrong card.

To illustrate the dynamics encountered under the most likely types of behavioral model ($B_{w_{1},w_{2},w_{3}}^{f}$ in the no-switch condition and $B_{w_{1},w_{2},w_{3}}^{r}$ in the switch condition) we have plotted the measured and modeled responses of a representative subject (#9), see Fig 10. The modeled response was averaged over posterior model probability (see top graphs of Figs 6 and 7). Note that for the selected subject only the $B_{\;w_{3}}^{f}$ (in the no-switch condition) and $B_{w_{2}}^{r}$ (in the switch condition) have posterior model probabilities close to one and therefore contributed to the shown modeled responses. Importantly, one can see that the expected model responses appropriately track the subject’s responses in all six experimental blocks, and that the deviations of the subject’s responses from the expected response are mostly explained by the response variability, as indicated by the shaded area.

## Discussion

We have used a probabilistic variant of the Wisconsin card sorting task (WCST) with belief solicitation to show that, in a rather complex environment, update of beliefs is modulated by an attentional focus mechanism. We analyzed behavioral data of 22 subjects using a meta-Bayesian framework [49,50]. This framework allowed us to compare multiple behavioral models, each implementing different assumptions about the underlying mechanisms that govern update of beliefs. We found evidence that incorporating an attentional focus mechanism within the behavioral model is the essential feature for modeling behavior. Specifically, we demonstrated that the attentional focus mechanism modulates subjects’ expectations about the relevance of each visual feature and consequently influences the update of beliefs when new visual evidence is provided. In addition, we found that introducing a deviation from optimal responses (as predicted by Bayesian decision theory), during belief solicitation, further increased model evidence in one experimental condition.

### WCST and belief solicitation

The variant of the WCST used here can be seen as a simple but representative task to which humans are often exposed, namely making decisions in situations where the relevant features of the environment are not obvious but need to be inferred first. What makes the WCST simpler when compared to natural environment is the reduced number of possible pre-learned hypotheses. However, the dynamic complexity is comparable to real world situations: (i) the rules of the environment can change, and (ii) in the specific WCST used here the experimenter occasionally ‘makes a mistake’ just as in the natural environment one often cannot know something with certainty. For the WCST task, these two naturally occurring sources of uncertainties make the necessary inference sufficiently complex to compute the subject’s uncertainty about the relevance of visual features. To better infer the hidden internal beliefs and uncertainties of subjects, we used belief solicitation in a form of a betting assignment, which reflect a subject’s hidden beliefs over the space of possible hypotheses. To our knowledge, such belief solicitation was not previously used in a WCST task, although similar experimental designs were used for simpler tasks [11,79].

### Modeling effects of attention on evolution of beliefs

To incorporate attentional-focus within the perceptual part of the behavioral model we modeled the dynamics of the hidden states of a probabilistic generative model with a winner-take-all (WTA) dynamics. This is a well-known type of dynamics applied to artificial neural networks [37–40,80–82] and used as a part of connectionist models of decision making and planning [19,25]. In addition, WTA network dynamics have been reported to capture a wide range of experimental findings [48,83–86].

For our purposes, the WTA neuronal network implemented a dynamic and self-regulated attention formation at the top level of a hierarchical representation of environmental features.

In comparison to the classical connectionist approach, e.g. [25], the main advantage of using the WTA dynamics within a Bayesian framework is that the adaptive coupling between the intrinsic network dynamics and external input (see Eqs (11) and (12)) is derived automatically as part of the update equations. These update equations provide Bayes-optimal behavior of the model by setting the connection weights to their optimal value. Although the optimization technique used by the brain may be different, such weight optimization may be assumed as a guiding computational principle of information processing in the brain.

Our finding—that competitive inhibitory WTA dynamics as a model of attentional focus is required for describing the hidden update process of subjects’ beliefs—is in agreement with previous findings of Wilson and Niv [1]. This suggests that in a WCST task humans actively track only the evidence corresponding to features they pay attention to, that is, the ones they found potentially relevant for the current task. Importantly, as a safe-guard against over-fitting the data with a complex WTA dynamics, we employed simpler (with a reduced number of free parameters) variants of the perceptual model. The fact that the less complex behavioral models have lower model evidence suggests that the WTA dynamics has indeed adequate complexity to describe the behavioral data.

### Predicting effects on behavior

The WTA dynamics introduces the following features in the evolution of beliefs: (i) faster convergence of beliefs to the working hypothesis; (ii) the beliefs are more inert to frequent changes in the environment, that is, to switch between the hypotheses sufficient amount of contradicting evidence has to accumulate. (iii) The beliefs change faster if the changes in the environment are rare, as after the fixed point is reached beliefs do not evolve further. In contrast, the diffusive dynamics of the SFM variants of the perceptual model is not bounded within finite volume of the belief space. Hence, as the posterior beliefs about a hypothesis’ relevance can be strongly separated if the environment is stable for a long period of time and, once the switch occurs it would take a very long time to adjust the beliefs as nothing constrains the separation of the posterior expectations.

Consequently, as our results suggest, the proposed attractor dynamics modulate expectations. This would predict the following effects on behavior: (i) Even small amount of evidence can have a big impact on beliefs, (ii) if changes in the environment are too frequent they will have smaller impact on beliefs than expected from the diffusive dynamics, and (iii) if changes in the environment are rare it will take less contradicting evidence to change the working hypothesis than predicted by the diffusive and unconstrained dynamics.

### Sub-optimality in human behavior

Although various studies have demonstrated that human behavior can approximate a Bayesian observer [26–28,60–62,87], human subjects can also behave sub-optimally when exposed to sufficiently complex tasks [28].

In recent work Acerbi et al. [51] have demonstrated that the response variability (deviation from expected response) is proportional to posterior uncertainty. Such a deviation from optimal responses can be explained if one assumes a stochastic representation of the posterior beliefs by the human brain [52,53].

Thus, to account for potential dependence of response variability on posterior uncertainty we considered two variants of the response model. In the first variant we assume that the response variability is constant over an experimental block. In the second variant we additionally allow for the variability of the modeled responses proportional to the posterior uncertainty (see Eqs (15) and (16)), which accounts for the potential stochastic representation of posterior beliefs.

Depending on the experimental condition both variants of the response model provide good accounts for the deviation of subjects’ responses from the optimal response. In the no-switch condition (the relevance of visual feature is unchanged during the block, see Fig 8H) we found that the response variability is indeed proportional to the posterior uncertainty; in the switch condition (Fig 8G) the evidence is inconclusive although in favor of the assumption that the response variability is fixed and independent of the posterior uncertainty. A reason for this inconclusive result may be the increased difficulty of the experimental task in the switch condition. An increased difficulty makes the behavioral responses noisier (responses deviate more from the optimal response compared to the no-switch condition, see Fig 10). As the average response variability increases, there is less information about the dependency of response variability on experimental trials. Hence, most of this additional variability may be explained simply by a rather high but constant level of response noise as formulated in the reduced response model.

### Related work on the computational role of attentional processes

Earlier work on the computational role of attention in the processing of sensory information suggested that attention can be understood as prior expectations about the sensory stimuli [88,89]. This rather simple view of attention as a prior has recently been extended to account for both selective and integrative attentional phenomena [34–36]. This extended view suggests that due to the computational complexity of the exact probabilistic inference and the limited amount of available cognitive resources, the human brain has to rely on approximations to efficiently solve perceptual tasks. In other words, the role of attention is to assign limited cognitive resources to the relevant part of the sensory stimuli, which provides local refinement of the internal representation of the hidden states of the environment.

However, this view on attention as an approximation to the exact Bayesian inference has been recently challenged. Under the free-energy principle [90]—which suggests that perception, attention, and action are all aimed toward suppressing the perceptual surprise about future sensory stimuli—attention is viewed as a sampling of only those parts of sensory stimuli that have high-precision in relation to the predictions of the internal model of the world [33]. Importantly, if the model of the world also predicts the precision of different parts of sensory stimuli, then that prediction is what Friston and colleagues propose to be associated with attention.

Our work presented here can be related to both assumptions about the computational role of attention, and as such cannot reconcile this dispute. Note, that the competitive attractor dynamics can be seen both as an approximation to the exact inference (the attractor dynamics regulates the update of beliefs by assigning the computational resources only to the most relevant hypothesis) and as a suppressor of the perceptual surprise (the attractor dynamics actively reduces the uncertainty about future sensory stimuli by predicting both the future expectation and precision of a categorical probability of hypothesis relevance; see Eq (9)).

### Potential limitations of the experimental design

We believe that the probabilistic WCST provides a promising experimental paradigm for investigating complex behavioral models. However, one can probably improve on the current design using two changes. Firstly, in spite of the initial training, several subjects exhibit rather poor performance in the no-switch condition (see Fig 2). Ten out of twenty two subjects show poor performance in at least one experimental block of the no-switch condition. Importantly, we have included these subjects in our analysis, because the model comparison did not show any correlation between subjects’ performance and the best fitting behavioral model. Also note that a key strength of the proposed model is that it can explain this poor performance well, see for example Fig 10; insofar a potentially suboptimal performance does not pose a limitation to the proposed modelling approach. However, the obtained results may be even more compelling if subjects practiced the task until a stable performance is reached for both conditions. Secondly, as mentioned in the Methods section, the error rate *ε* was set to values that induced the most distinct behavioral responses between two experimental conditions, while rendering the switch condition informative enough to induce betting responses in subjects. However, these led to a partially imbalanced manipulation between conditions. Thus, a potential improvement would be to introduce a fractal design, such that both the error rate and the switch probability are incrementally increased. Such a fractal design would provide further insights into how each environmental parameter influences behavior and what effects, if any, each parameter might have on the model comparison.

### Limitations of the analytical method

Similar to the experimental design, the analytical approach presented here may also be potentially improved upon. Firstly, as mentioned in the Methods section, the behavioral model proposed here is not the only possible formulation. Depending on how one defines the observation likelihood (Eq (4)) and the parametrization of the hypothesis probability (Eq (5)), one can obtain different variants of the perceptual model. Although we have tested a couple of them (one additional, alternative formulation is described in S1 Text), there is a large number of possible perceptual models. We anticipate that more studies are required to come to a general conclusion which of the models or model families is the most useful for describing behavioral data of studies similar to the one presented here. Secondly, the model comparison presented here relies solely on the Bayesian model selection that is useful for inferring which of the given models is most likely to generate the data. However, it cannot be directly used to answer the question whether a given model is a good predictor of behavior. To address this question one has to rely on cross-validation strategies, that is, on model testing [91]. Still, one important prior assumption of model testing is that the behavior can be described by parameters which are stable over blocks. We do not assume that this is the case for our experimental data as subjects were not over-trained which would motivate the assumption that subjects performed the task in some stable parameter regime. Thus, it is plausible that the experience in previous experimental blocks influences, at least slightly, the behavior in subsequent blocks. For this reason model testing may not be usefully applicable to our study. Nevertheless, for future studies changes to the training procedure may stabilize behavior across experimental blocks and would allow one to also apply model testing methods to predict behavior.

### Neuroimaging application

Although the presented analysis has been applied to behavioral data only, it would be potentially useful and feasible to extend the behavioral analysis to the investigation of neuroimaging data. The inferred belief trajectories would be used as regressors [13], and thus can provide insights into the functional aspects of specific brain areas involved in the decision making process during the ongoing task.

### Conclusion

We found strong evidence that an attention-like mechanism modulates the update of beliefs in human subjects who had to infer the relevance of various features in a dynamic and noisy environment. Effectively, this attentional focus facilitates the increase of expectations about the relevant feature and inhibits the expectations about irrelevant features. Subsequently, these modulated expectations affect update of beliefs. We expect that the same computational mechanism can be applied to modelling other complex tasks that impose high cognitive load on subjects, thus require the attentional focus strategies for decision making.

## Supporting Information

S1 Text An alternative formulation of the perceptual model. Contains derivations of an alternative perceptual model (and reduced model variants) and also contains the results of the model comparison.
(PDF)

S2 Text Response model derivation. Contains detailed derivation of the response model within the framework of Bayesian decision theory.
(PDF)

S1 Data Collection of data files. Contains behavioral data, posterior and prior expectation (and covariance matrix) of the free model parameters, estimated log-model evidence for each behavioral model, and the model comparison results.
(GZ) Click here for additional data file.

S1 Fig Correlations between the expected model performance and the measured subjects’ performance. Pearson correlation coefficient *r*_*corr*_ between the mean subject performance and the mean model performance, for each behavioral model in the switch (top) and no-switch condition (bottom).
(TIFF)
