# Supplementary material for: Modeling the Evolution of Beliefs Using an Attentional Focus Mechanism
Source: PLoS Comput Biol. 2015 Oct 23;11(10):e1004558. doi: 10.1371/journal.pcbi.1004558 (PMC4619749; doi:10.1371/journal.pcbi.1004558)
Supplement: S1 Text — Contains derivations of an alternative perceptual model (and reduced model variants) and also contains the results of the model comparison. (PDF) [file pcbi.1004558.s001.pdf]

An alternative formulation of the hierarchical perceptual model

Generative model

For the formulation of the alternative generative model we will make the same assumptions as before, namely that beliefs and sensory information are combined in a Bayes-optimal fashion and that the update of beliefs can be represented as Markov process.

In this formulation we will describe the probabilistic WCST as a hierarchical hypothesis comparison task. Assuming that the ith ($i \in \{1,2,3\}$) visual feature and that the jth exemplar (exemplars are encoded either as 0 or 1, thus $j \in \{0,1\}$) are currently relevant for the selection process, the probability of selecting a card containing the relevant exemplar is defined as

$$p(\vec{e}|E_t = j, F_t = i) = (1-\varepsilon)^{\delta_{e_i,j}} \varepsilon^{\delta_{e_i,1-j}}, \tag{1}$$

where $\delta_{x,y}$ denotes Kronecker's delta. In other words, the ith component of the observation vector $\vec{e} \in \{0,1\}^3$, will be equal to $e_i = j$ (jth exemplar will be selected) with probability $1-\varepsilon$ or $e_i = 1-j$ (the oposite exemplar will get selected) with probability ε.

The probability that one of the exemplars is currently relevant is defined as

$$P(E_t|F_t = i) = \varphi\left(x_{t,i}^{(e)}\right)^{E_t^{(i)}} \left(1-\varphi\left(x_{t,i}^{(e)}\right)\right)^{1-E_t^{(i)}}, \tag{2}$$

where $\varphi(x) = \frac{1}{1+e^{-x}}$. Here $\vec{x}_t^{(e)}$ denotes 3D state space vector which encodes the conditional probabilities that one of the exemplars is currently relevant given that the ith visual feature is relevant for the selection process. We will define the time evolution of the state space vector as a diffusive process

$$\vec{x}_{t+1}^{(e)} = \tau_e \vec{x}_t^{(e)} + \vec{\omega}_t^{(e)}, \tag{3}$$

where $\vec{\omega}_t^{(e)}$ denotes a vector of *i.i.d* random variables drawn from a Gaussian distribution $\mathcal{N}\left(\vec{\omega}_t^{(e)}; 0, q_e I_3\right)$, and τ_e denotes a time constant. The transition probability of the state space vector $\vec{x}_t^{(e)}$ is given as

$$p\left(\vec{x}_t^{(e)}|\vec{x}_{t-1}^{(e)}\right) = \mathcal{N}\left(\vec{x}_t^{(e)}; \tau_e \vec{x}_{t-1}^{(e)}, q_e I_3\right) \tag{4}$$

We will define the probability of a visual feature's relevance as a categorical distribution, hence

$$P(F_t|\vec{x}_t^{(f)}) = \prod_{i=1}^{3} \pi_i\left(\vec{x}_t^{(f)}\right)^{\delta_{F_t,i}}, \tag{5}$$

where $\pi_i(\vec{x}) = \frac{e^{x_i}}{\sum_{j=1}^{3} e^{x_j}}$. Similar to Eq. (2), $\vec{x}_t^{(f)}$ encodes the relevance of visual features. Here, we will use a general functional form of the WTA dynamics to define the time evolution of $\vec{x}_t^f$, thus

$$\vec{x}_t^f = \vec{g}(\vec{x}_{t-1}^{(f)}) = \tau_f \vec{x}_{t-1}^{(f)} + \kappa_f + W_{lat}\vec{\varphi}\left(\vec{x}_{t-1}^{(f)}\right) + \vec{\omega}_t^{(f)}, \tag{6}$$

where τ_f denotes time constant, κ_f defines the attractor point, $\vec{\omega}_t^{(f)} \sim \mathcal{N}\left(\vec{\omega}_t^{(f)}; 0, q_f I_3\right)$, and $\vec{\varphi}(\vec{x}) = (\varphi(x_1), \varphi(x_2), \varphi(x_3))^T$. As before we use W_{lat} to denote the connectivity matrix of the symmetric inhibitory connections, thus

$$[W_{lat}]_{i,j} = \begin{cases} -w, & \text{if} \quad i \neq j \\ 0, & \text{if} \quad i = j \end{cases}, \; w = 2\kappa_f. \tag{7}$$

Hence, the transition probability of the state space vector $\vec{x}_t^{(f)}$ is given as

$$p\left(\vec{x}_t^{(f)} | \vec{x}_{t-1}^{(f)}\right) = \mathcal{N}\left(\vec{x}_t^{(f)}; \vec{g}\left(\vec{x}_{t-1}^{(e)}\right), q_f I_3\right). \tag{8}$$

Given the observation likelihood (1), the exemplar probability (2), the feature probability (5), and the transition probabilities (4), (8) we write the full generative model as

$$p\left(\vec{e}_t, E_t, F_t, \vec{x}_t^{(e)}, \vec{x}_t^{(f)}, \vec{x}_{t-1}^{(e)}, \vec{x}_{t-1}^{(f)} | e_{1\dots t-1}\right) = \tag{9}$$
$$p\left(\vec{e}_t, E_t, F_t | \vec{x}_t^{(e)}, \vec{x}_t^{(f)}\right) p\left(\vec{x}_t^{(e)}, \vec{x}_t^{(f)}, \vec{x}_{t-1}^{(e)}, \vec{x}_{t-1}^{(f)} | e_{1\dots t-1}\right),$$

where

$$p\left(\vec{e}_t, E_t, F_t | \vec{x}_t^{(e)}, \vec{x}_t^{(f)}\right) = \tag{10}$$
$$\prod_{i=1}^{3} \left[p\left(\vec{e}_t | E_t, F_t\right) p\left(E_t | F_t, x_{t,F_t}^{(e)}\right) p\left(F_t | \vec{x}_t^{(f)}\right)\right]^{\delta_{F_t,i}}.$$

Variational inference

To obtain the update equations for the posterior probability of the hidden states, $p\left(E_t, F_t, \vec{x}_t^{(e)}, \vec{x}_t^{(f)} | e_{1\dots t}\right)$, we require a compact form of the generative model obtained by integrating out $\vec{x}_{t-1}^{(e)}$ and $\vec{x}_{t-1}^{(f)}$ from (9), that is,

$$p\left(\vec{x}_t^{(e)}, \vec{x}_t^{(f)} | e_{1\dots t-1}\right) = \tag{11}$$
$$\int_{-\infty}^{\infty} \int_{-\infty}^{\infty} \left(\vec{x}_t^{(e)}, \vec{x}_t^{(f)}, \vec{x}_{t-1}^{(e)}, \vec{x}_{t-1}^{(f)} | e_{1\dots t-1}\right) \mathrm{d}\vec{x}_{t-1}^{(e)} \mathrm{d}\vec{x}_{t-1}^{(f)}.$$

Hence, the predictive probabilities can be written as

$$p\left(\vec{x}_t^{(e)}|e_{1\ldots t-1}\right) = \mathcal{N}\left(\vec{x}_t^{(e)}; \tau_e\vec{\mu}_{t-1}^{(e)}, \tau_e^2\Sigma_{t-1}^{(e)} + q_e I_3\right), \tag{12}$$

$$p\left(\vec{x}_t^{(f)}|e_{1\ldots t-1}\right) = \mathcal{N}\left(\vec{x}_t^{(f)}; \vec{g}\left(\vec{\mu}_{t-1}^{(f)}\right), \tilde{\Sigma}_{t-1}^{(f)} + q_f I_3\right), \tag{13}$$

where $\tilde{\Sigma}_{t-1}^{(f)} = \partial_{\vec{x}^{(f)}}\vec{g} \cdot \Sigma_{t-1}^{(f)} \cdot \partial_{\vec{x}^{(f)}}\vec{g}^T\big|_{\vec{x}^{(f)}=\vec{\mu}_{t-1}^{(f)}}$. The vectors $\vec{\mu}_{t-1}^{(e)}$, $\vec{\mu}_{t-1}^{(f)}$ and covariance matrices $\Sigma_{t-1}^{(e)}$, $\Sigma_{t-1}^{(f)}$ denote the posterior expectations and uncertainties, respectively, from the previous time step.

We will apply a mean field approximation to the posterior probability of hidden states, thus

$$p\left(E_t, F_t, \vec{x}_t^{(e)}, \vec{x}_t^{(f)}|e_{1\ldots t}\right) \approx q(F_t)q(\vec{x}_t^{(f)})\prod_{i=1}^{3} q(x_{t,i}^{(e)})q(E_t^i), \tag{14}$$

where

$$q(E_t^i) = \left[\rho_{t,i}^{(e)}\right]^{E_t^i}\left[1-\rho_{t,i}^{(e)}\right]^{1-E_t^i}, \tag{15}$$

$$q(x_{t,i}^{(e)}) = \mathcal{N}\left(x_{t,i}^{(e)}; \mu_{t,i}^{(e)}, \sigma_{t,i}^{(e)}\right), \tag{16}$$

$$q(F_t) = \prod_{i=1}^{3}[\rho_{t,i}^{(f)}]^{\delta_{F_t,i}}, \tag{17}$$

$$q(\vec{x}_t^{(f)}) = \mathcal{N}\left(\vec{x}_t^{(f)}; \vec{\mu}_t^{(f)}, \Sigma_t^{(f)}\right). \tag{18}$$

We have followed the same method, as described in the main article, to obtain the update equations for the parameters of the marginal posterior distributions shown above. As we will not go in details again how to obtain the update equations from the variational energy, we will just present the update equation for each

parameter of the approximate posterior:

$$\rho_{t,i}^{(e)} = \varphi\left(\rho_{t-1,i}^{(f)} \cdot \left[\tau_e \mu_{t-1}^{(e)} + \ln \frac{p\left(\vec{e}|E_t^i = 0\right)}{p\left(\vec{e}|E_t^i = 1\right)}\right]\right), \tag{19}$$

$$\mu_{t,i}^{(e)} = \tau_e \vec{\mu}_{t-1}^{(e)} + \sigma_{t,i} \rho_{t-1,i}^{(f)} \left[\rho_{t,i}^{(e)} - \varphi\left(\tau_e \vec{\mu}_{t-1}^{(e)}\right)\right], \tag{20}$$

$$\sigma_{t,i}^{(e)} = \frac{\tau_e^2 \sigma_{t-1,i}^{(e)}}{1 + \rho_{t-1,i}^{(f)} \tau_e^2 \sigma_{t-1,i}^{(e)} \varphi\left(\tau_e \vec{\mu}_{t-1}^{(e)}\right)\left(1 - \varphi\left(\tau_e \vec{\mu}_{t-1}^{(e)}\right)\right)}.$$

$$\rho_{t,i}^{(f)} = \frac{e^{h\left(e_i, \rho_{t,i}^{(e)}, \mu_{t,i}^{(e)}, \sigma_{t,i}^{(e)}\right) + \tilde{\mu}_{t,i}^{(f)}}}{\sum_{j=1}^{3} e^{h\left(e_j, \rho_{t,j}^{(e)}, \mu_{t,j}^{(e)}, \sigma_{t,j}^{(e)}\right) + \tilde{\mu}_{t,j}^{(f)}}}, \tag{21}$$

$$\mu_{t,i}^{(f)} = \vec{g}\left(\vec{\mu}_{t-1}^{(f)}\right) + \Sigma_t^{(f)}\left[\vec{\rho}_t^{(f)} - \vec{\pi}\left(\vec{g}\left(\vec{\mu}_{t-1}^{(f)}\right)\right)\right], \tag{22}$$

$$\Sigma_t^{(f)} = \left[I_3 + \tilde{\Sigma}_t^{(f)} Y\right]^{-1} \tilde{\Sigma}_t^{(f)}, \tag{23}$$

where

$$Y = \bigoplus_{i=1}^{3} \pi_i\left(\vec{g}\left(\vec{\mu}_{t-1}^{(f)}\right)\right) - \vec{\pi}\left(\vec{g}\left(\vec{\mu}_{t-1}^{(f)}\right)\right) \cdot \vec{\pi}\left(\vec{g}\left(\vec{\mu}_{t-1}^{(f)}\right)\right)^T,$$

$$\begin{aligned} h\left(e_i, \rho_{t,i}^{(e)}, \mu_{t,i}^{(e)}, \sigma_{t,i}^{(e)}\right) &= \rho_{t,i}^{(e)} \ln \varepsilon + (1 - \rho_{t,i}^{(e)}) \ln(1 - \varepsilon) \\ &+ e_i \ln \frac{\varepsilon}{1 - \varepsilon}\left(1 - 2\rho_{t,i}^{(e)}\right) \\ &+ \rho_{t,i}^{(e)} \mu_{t,i}^{(e)} - k_i. \end{aligned}$$

We used spherical radial approximation [1] to compute the following expectation term

$$\begin{aligned} k_i &= \int \ln(1 + e^x) \mathcal{N}\left(x; \mu_{t,i}^{(e)}, \sigma_{t,i}^{(e)}\right) \mathrm{d}x \\ &\approx \frac{1}{2}\left[\ln\left(1 + e^{\mu_{t,i}^{(e)} + \sqrt{\sigma_{t,i}^{(e)}}}\right) + \ln\left(1 + e^{\mu_{t,i}^{(e)} - \sqrt{\sigma_{t,i}^{(e)}}}\right)\right] \end{aligned}$$

Variants of the perceptual model

The above presented equations describe the full perceptual model with the following set of free parameters

$$\gamma = \{\varepsilon, \tau_{e,f}, q_{e,f}, \kappa_f, \sigma_0^{(e)}, \sigma_0^{(f)}\} \tag{24}$$

We will consider that the initial prior expectation are always fixed, thus $\mu_0^{(e)}, \mu_0^{(f)} = 0$.

To obtain the structure-free variant of the perceptual model we will remove the attractor dynamics form the top layer of the hierarchy by setting $\kappa_f = 0$.

Non-bayesian formulation

Similar to the derivations in the main text, we will here also use the Bayesian formulation of the update equations to define the non-Bayesian update equations. We obtain this update equations by fixing updates of the posterior expectations and fixing prior beliefs about exemplar and feature probability, thus

$$\rho_{t,i}^{(e)} = \varphi\left(\frac{1}{3}\ln\frac{p\left(\vec{e}|E_t^i = 0\right)}{p\left(\vec{e}|E_t^i = 1\right)}\right), \tag{25}$$

$$\mu_{t,i}^{(e)} = \tau_e\vec{\mu}_{t-1}^{(e)} + \frac{\alpha^{(e)}}{3}\left[\rho_{t,i}^{(e)} - \varphi\left(\tau_e\vec{\mu}_{t-1}^{(e)}\right)\right], \tag{26}$$

$$\rho_{t,i}^{(f)} = \frac{e^{h\left(e_i,\rho_{t,i}^{(e)},\mu_{t,i}^{(e)},0\right)}}{\sum_{j=1}^{3} e^{h\left(e_j,\rho_{t,j}^{(e)},\mu_{t,j}^{(e)},0\right)}}, \tag{27}$$

$$\mu_{t,i}^{(f)} = \vec{g}\left(\vec{\mu}_{t-1}^{(f)}\right) + \alpha^{(f)} I_3\left[\vec{\rho}_t^{(f)} - \vec{\pi}\left(\vec{g}\left(\vec{\mu}_{t-1}^{(f)}\right)\right)\right]. \tag{28}$$

The full set of parameters for the non-Bayesian perceptual model is

$$\gamma = \{\varepsilon, \tau_{e,f}, \kappa_f, \alpha^{(e)}, \alpha^{(f)}\}. \tag{29}$$

Same as in the main text, we will obtain the structure-free form of the perceptual model by setting $\kappa_f = 0$

Response model

For the response model we will use the same formulation as in the main text. For the Bayesian variants of the perceptual model we will consider two types of response model: the reduced response model $\theta = \{\theta_1, \theta_3\}$, and the full response model $\theta = \{\theta_1, \theta_2, \theta_3\}$. In the case of the non-Bayesian variants of the perceptual model we will only consider the reduced version of the response model.

Model comparison

To summarize, the full comparison set consists of the following models

$$M \in \{BM, N\hat{B}_d^r, N\hat{B}_w^r, \hat{B}_d^r, \hat{B}_w^r, \hat{B}_d^f, \hat{B}_w^f\},$$

where, as before, BM denotes the baseline model, $\hat{NB}$ denotes the non-Bayesian and $\hat{B}$ denotes the Bayesian variants of the perceptual model. The superscript denotes the type of the response model ($f \rightarrow$ full, $r \rightarrow$ reduced), and the subscript denotes the type of the connectivity matrix ($d \rightarrow$ without inhibition, $w \rightarrow$ with inhibition)

Results

Following the same procedure for model comparison as in the main text we present the direct comparison of behavioural models in Fig. (1) and Fig. (2). In the switch condition the structure-free variant of the non-Bayesian perceptual model provides a highly likely description of behaviour (XP is only slightly below the confidence threshold level). Surprisingly, in the no-switch condition the model comparison suggests that the baseline model (constant expectations) is the best fit to behaviour (XP is again only slightly below the confidence threshold level). Similar conclusions can be made using a family-wise model comparison presented in Fig. (3 A-F), as only the model families which contain either baseline model (BM, RR) or the structure free variant of the non-Bayesian perceptual model (NB, RR) have a high XP. However, the fact that the baseline model actually has high model evidence when compared to the dynamical models, suggests that this alternative formulation of the generative model provides a poor account for the measured behaviour. This is confirmed by a family wise model comparison between the alternative (described here, $I = \{\hat{NB}^{r}_{d,w}, \hat{B}^{r,f}_{d,w}\}$) and the original formulation (described in the main text, $II = \{NB^{r}_{d,w_1,w_2,w_3}, B^{r,f}_{d,w_1,w_2,w_3}\}$) of the perceptual model (Fig. (3 G-H)). The comparison of the two families of generative models demonstrates that the original formulation (as presented in the main text) of the generative model is more likely to generate the data (high XP in both experimental conditions). From these results we can conclude that the original formulation of the perceptual model is closer to the true mapping of observations to beliefs, which further suggests that the conditional probabilities (e.g. the probability of selecting an exemplar given that the ith visual feature is relevant) are not explicitly represented in the brain, but rather only implicitly captured by the interactions in the belief space (*e.g.* connectivity matrix).

References

[1] Ienkaran Arasaratnam and Simon Haykin. Cubature kalman filters. *Automatic Control, IEEE Transactions on*, 54(6):1254–1269, 2009.

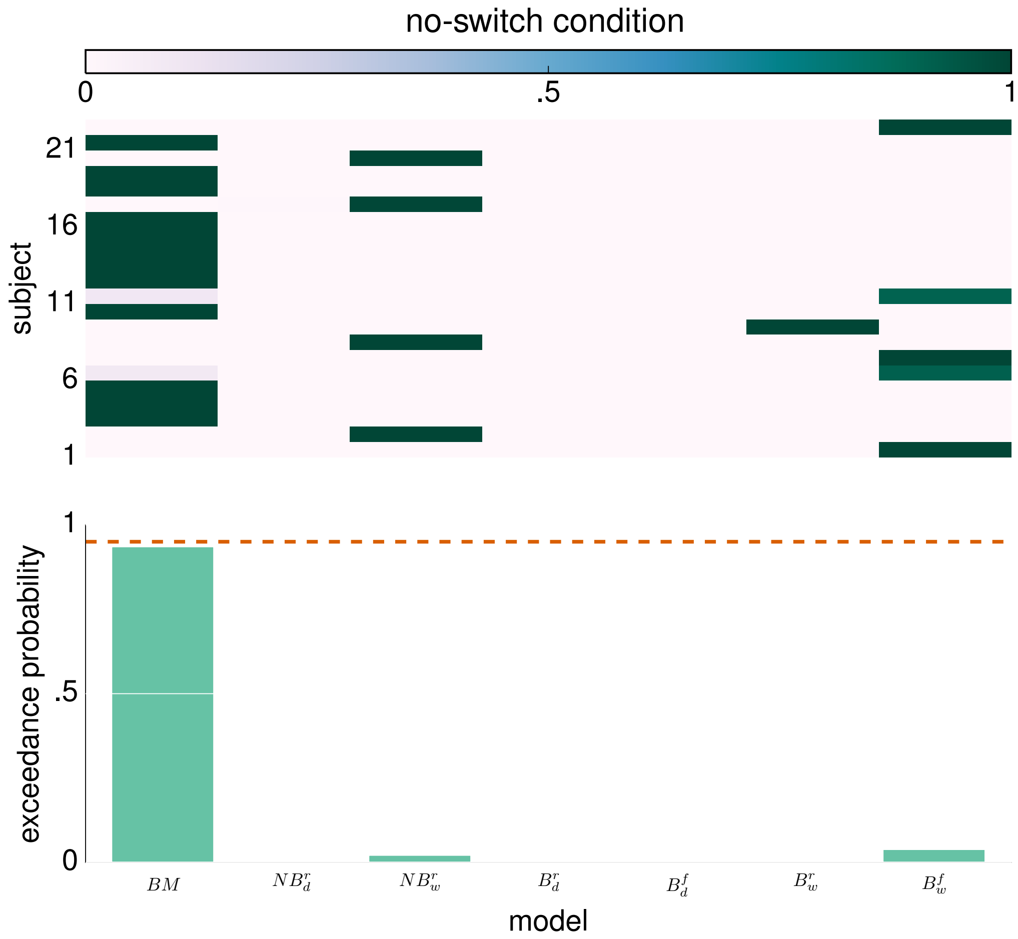

Figure 1: Random-effects model comparison for the no-switch condition. (top) Model probability (see color bar) for each subject. (bottom) Exceedance probability (XP) that a given model is more likely to generate the data than any other model (see main text for description). The dashed orange line denotes the confidence threshold level set at 0.95.

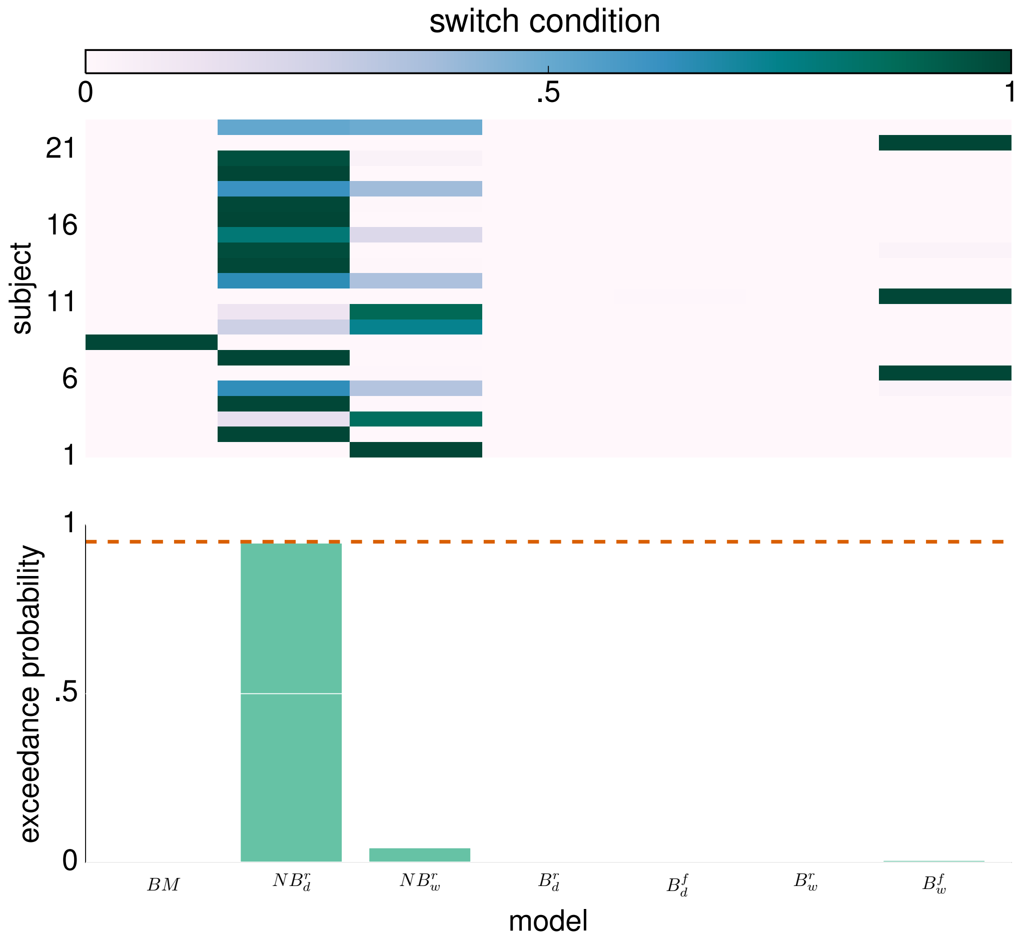

Figure 2: Random-effects model comparison for the switch condition. (top) Model probability (see color bar) for each subject. (bottom) Exceedance probability (XP) that a given model is more likely to generate the data than any other model (see main text for description). The dashed orange line denotes the confidence threshold level set at 0.95.

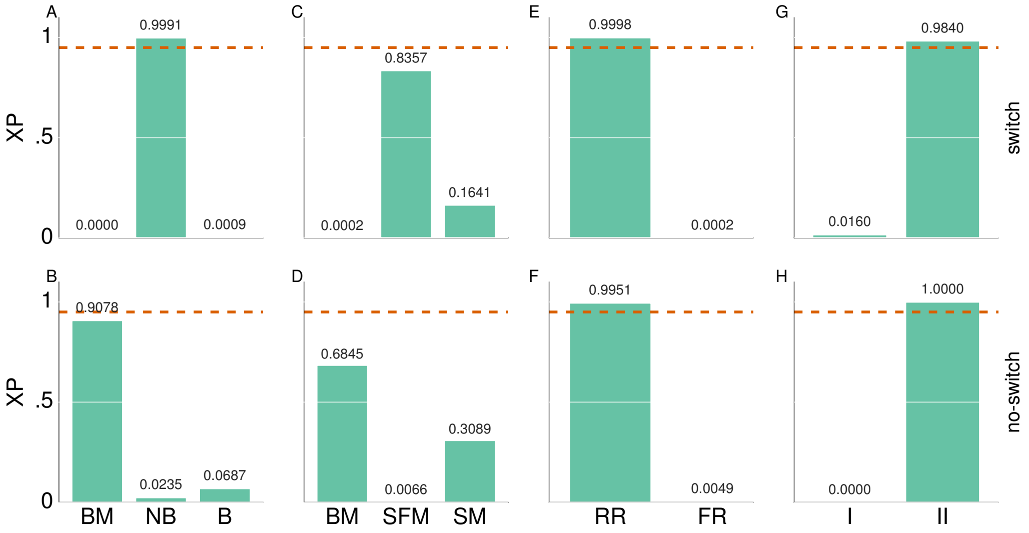

Figure 3: Family-wise model comparisons. Top graphs show the exceedance probability of model families in the switch condition; the bottom graphs show the exceedance probability of the model families in the no-switch condition (see text for details about the definition of each model family). The dashed orange line denotes the confidence threshold level set at 0.95.
