# Supplementary material for: Modeling the Evolution of Beliefs Using an Attentional Focus Mechanism
Source: PLoS Comput Biol. 2015 Oct 23;11(10):e1004558. doi: 10.1371/journal.pcbi.1004558 (PMC4619749; doi:10.1371/journal.pcbi.1004558)
Supplement: S2 Text — Contains detailed derivation of the response model within the framework of Bayesian decision theory. (PDF) [file pcbi.1004558.s002.pdf]

# Optimal gambling responses in the probabilistic WCST

From the observed sequence of experimenter's choices $e_{1\ldots t}$ subjects have to estimate a posterior probability about the visual feature relevance. Let us denote with $p_i = P(F = i|e_{1\ldots t})$ such posterior probability of the $i$th feature (color = 1, motion = 2, shape = 3).

Subjects were instructed that at the and of the experiment, one of the trials will be randomly selected and they will obtain the amount of money they assigned to the truly relevant feature. Let $r_i \in [0,1]$ be the fraction of money that subjects decide to assign to $i$th feature. To define the utility of such a gamble we will assume that subjects' absolute risk aversion has the form of the hyperbolic absolute risk aversion (HARA) formulated as

$$A(x) = \frac{\alpha}{\theta \alpha x + \beta}, \tag{1}$$

where $\theta$, $\alpha > 0$, $\beta$ is a real valued constant, and $x$ denotes wealth.

HARA is often used in models of financial decision making [1, 2, 3] as it is one of the most general classes of analytic utility functions. HARA refers to a property of utility functions of final wealth (von Neumann-Morgenstern utility functions [4]), which assume that the wealthier a person is the less value he puts to a fixed monetary gain [5] (e.g. one euro will be more valuable to someone who has only one euro than someone who has millions of euros).

The absolute risk aversion is defined as a negative ratio of risk attitude (second derivative of the utility function) and utility density (first derivative of the utility function), thus

$$A(x) = -\frac{U''(x)}{U'(x)} = -\frac{\mathrm{d}}{\mathrm{d}x} \ln U'(x). \tag{2}$$

Hence, the corresponding HARA utility function has the following form

$$U(x) = \frac{(\alpha x - x_s)^{1-\frac{1}{\theta}}}{1-\frac{1}{\theta}}, \tag{3}$$

where $x_s = -\frac{\beta}{\theta}$.

Applying the HARA utility to our gambling task we get

$$\tilde{U}(r_1, r_2|F) = \begin{cases} \frac{(\alpha r_1 - x_s)^{1-\frac{1}{\theta}}}{1-\frac{1}{\theta}}, & \text{if} \quad F = 1 \\ \frac{(\alpha r_2 - x_s)^{1-\frac{1}{\theta}}}{1-\frac{1}{\theta}}, & \text{if} \quad F = 2 \\ \frac{(\alpha r_3 - x_s)^{1-\frac{1}{\theta}}}{1-\frac{1}{\theta}}, & \text{if} \quad F = 3 \end{cases} \tag{4}$$

where $F$ denotes the visual feature and $r_3 = 1 - r_1 - r_2$. Given the posterior beliefs $\vec{p}_t$ at trial $t$ the expected utility of any response $\vec{r}$ is obtained as

$$Q(\vec{r}, \vec{p}_t) = \sum_{i=1}^{3} U(r_i) p_{t,i}. \tag{5}$$

Bayesian decision theory (BDT) suggests that the optimal policy $\vec{r}^*$ is the one that maximises expected utility. Formally we write this as

$$\vec{r}^* = \arg\max_{\vec{r}} Q(\vec{r}, \vec{p}_t). \tag{6}$$

In other words, the response $\vec{r}^*$ is optimal if the following three conditions are satisfied:

- Partial derivatives $\partial_{r_1} Q$ and $\partial_{r_2} Q$ are zero at $\vec{r}^*$.
- Second order partial derivative $\partial_{r_1,r_1} Q(\vec{r}, \vec{p}_t)|_{\vec{r}=\vec{r}^*} < 0$
- Determinant of the Hessian matrix is larger then zero, that is

$$|H| = \left(\partial_{r_1,r_1} Q \cdot \partial_{r_2,r_2} Q - [\partial_{r_1,r_2} Q]^2\right)|_{\vec{r}=\vec{r}^*} > 0. \tag{7}$$

From the first constraint

$$\partial_{r_1} Q(\vec{r}, \vec{p}_t) = U'(r_1) p_{t,1} - U'(r_3) p_{t,3} \equiv 0,$$
$$\partial r_2 Q(\vec{r}, \vec{p}_t) = U'(r_2) p_{t,2} - U'(r_3) p_{t,3} \equiv 0,$$

we obtain the extremum of the expected utility $Q$ at $U'(r_i^*) = \frac{1}{p_{t,i}}$, that is, at

$$r_{t,i}^* = \frac{x_s + p_{t,i}^{\theta}}{\alpha}. \tag{8}$$

This point is indeed the maximum of the expected utility as both second order conditions are satisfied, that is,

$$\partial_{r_1,r_1} Q|_{\vec{r}=\vec{r}^*} = -\frac{\alpha^2}{\theta}\left(p_1^{-\theta} + p_2^{-\theta}\right) < 0, \forall\, \alpha, \theta > 0,$$
$$|H|_{\vec{r}=\vec{r}^*} = \frac{\alpha^4}{\theta^2}\left((p_1 p_2)^{-\theta} + (p_3 p_2)^{-\theta} + (p_1 p_3)^{-\theta}\right) > 0, \forall\, \alpha, \theta > 0.$$

Let us now further constrain the offset $x_s$ and normalization constant $\alpha$. The utility function $\tilde{U}$ should be real valued for any response $\vec{r}$. Thus, it has to hold that $\alpha r_i \geq x_s$. As the minimal fraction of money assigned to any visual feature

is zero then the offset $x_s$ has to be smaller than zero. In addition, we have a requirement that the optimal response $r_i*$ is constrained to $[0,1]$ interval, thus, $x_s \geq -p^{\theta}_{t,i}$. As this inequality has to be satisfied for all possible values of $p_{t,i}$ we obtain that $x_s \geq 0$. Thus, combining this with the previous constraint we get that $x_s = 0$, that is, $\beta = 0$. Finally, as we have defined the response as the fraction of money assigned to each visual feature we have to normalize the optimal response by setting $\alpha = \sum_{i=1}^{3} p^{\theta}_{t,i}$. Thus, we can write

$$r^*_{t,i} = \frac{e^{\theta m_{t,i}}}{\sum_{j=1}^{3} e^{\theta m_{t,j}}}, \tag{9}$$

where we have re-parametrized posterior beliefs in terms of the real valued parameter $m_i$, where $p_{t,i} \propto e^{m_{t,i}}$.

Note that the remaining free parameter $\theta$ is inversely related to the risk aversion. Risk aversion is defined as the second derivative of the utility function, thus

$$RA = U''(x) = -\frac{\alpha^2}{\theta} x. \tag{10}$$

A person is said to be risk-averse for $RA < 0$. As we considered $\theta$ only as a positive real valued parameter we implicitly assume that all subjects have risk-averse tendencies. However, this should not be considered a limitation here, because for $\theta < 0$ the above solution $r^*_i$ actually minimises the expected utility ($\partial_{r1,r1} Q > 0$). Thus, for $\theta < 0$ the optimal response corresponds to assigning all the money to the feature with the highest posterior belief $p_i$. As such a behaviour corresponds to the one obtained for the linear utility function, obtained in the limit $\theta \rightarrow$, one cannot distinguish between neutral risk attitude ($RA = 0$) and risk seeking attitude ($RA > 0$). Thus, it is safe to assume that setting $\theta$ to positive-definite values covers the full scope of expected behaviour.

# References


[1] Christopher D Carroll and Miles S Kimball. On the concavity of the consumption function. *Econometrica: Journal of the Econometric Society*, pages 981–992, 1996.

[2] Darrell Duffie, Wendell Fleming, H Mete Soner, and Thaleia Zariphopoulou. Hedging in incomplete markets with hara utility. *Journal of Economic Dynamics and Control*, 21(4):753–782, 1997.

[3] Geoffrey Kingston and Susan Thorp. Annuitization and asset allocation with hara utility. *Journal of Pension Economics and Finance*, 4(03):225–248, 2005.

[4] Ludwig Johann Neumann and Oskar Morgenstern. *Theory of games and economic behavior*. Princeton University Press Princeton, NJ, 1947.

[5] Kevin Roebuck. *Object-relational mapping (ORM): High-impact Strategies-What You Need to Know: Definitions, Adoptions, Impact, Benefits, Maturity, Vendors*. Emereo Publishing, 2012.
