# Supplementary material for: Modeling the Evolution of Beliefs Using an Attentional Focus Mechanism
Source: PLoS Comput Biol. 2015 Oct 23;11(10):e1004558. doi: 10.1371/journal.pcbi.1004558 (PMC4619749; doi:10.1371/journal.pcbi.1004558)
Supplement: S1 Data — Contains behavioral data, posterior and prior expectation (and covariance matrix) of the free model parameters, estimated log-model evidence for each behavioral model, and the model comparison results. (GZ) [file pcbi.1004558.s003.gz › README.pdf]

Behavioral data, posterior and prior parameter distributions, and model evidence

1 Behavioral data

"data alltrials.txt" contains the raw behavioral data:

- column 1 → trial number
- column 2 → switching (1) no-switching (0) experimental condition
- columns 3 - 8 → selection (1) or no-selection (0) of the corresponding exemplar
- column 9 → position of the selected card
- columns 10 - 12 → fraction of the money assigned to corresponding visual feature
- column 13 → position of the card containing the relevant stimulus
- column 14 → relevant exemplar
- column 15 → relevant visual feature
- column 16 → response times

2 Model parameters

The model fitting and model comparison was performed in Matlab R2013a. The result of the analysis are saved in the mat files which can be imported within a MATLAB workspace with "load" command or within a Python with "scipy.io.loadmat('file.mat')".

"posterior_and_evidence*.mat" contains all the relevant information for reconstructing the prior and the posterior parameter distributions and the corresponding model evidence. The results are saved within a cell array denoted with "vars". Each cell of 'vars' contains the information corresponding to a single subject. Thus, each cell is a 6x5 cell-matrix, where the rows of the matrix represent different experimental blocks. The information in columns is organized as follows: column 1 - posterior expectations of the free parameters (vector) column 2 - posterior covariance matrix (matrix) column 3 - prior expectations (vector) column 4 - prior covariance (vector containing only diagonal elements) column 5 - log-model evidence

The acronym 'NSW' denotes no-switch condition, 'SW' denotes switch condition, and 'ALT' denotes alternative perceptual model described in S1 Text.

2.1 Model parameters and their position within the data vectors

Given the vector of transformed free model parameters $\vec{\chi}$ (see section "List of models and model evidence computation" within the main article), then for each model the vector is organized as follows:

- $\vec{\chi}_{BM} = \left(\vec{\mu}_f^0, \ln \vec{\alpha}\right)$
- $\vec{\chi}_{NB^r_{rd}} = \left(\vartheta(\alpha), \vartheta(\tau_e), \mu_0^{(e)}, \vartheta(\epsilon), \vartheta(\theta_2), \vartheta(\theta_1)\right)$
- $\vec{\chi}_{NB^r_{rw}} = \left(\vartheta(\kappa^e), \vartheta(\alpha), \vartheta(\tau_e), \mu_0^{(e)}, \vartheta(\epsilon), \vartheta(\theta_2), \vartheta(\theta_1)\right)$
- $\vec{\chi}_{NB^r_d} = \left(\vartheta(w^{dist}), \vartheta(\theta_1), \vartheta(\alpha), \vartheta(\tau_{e,f}), \mu_0^{(e)}, \vartheta(\epsilon), \vartheta(\theta_2), \right)$
- $\vec{\chi}_{NB^r_{w_1}} = \left(\vartheta(\kappa^{e,f}), \vartheta(\alpha), \vartheta(\tau_{e,f}), \mu_0^{(e)}, \vartheta(\epsilon), \vartheta(\theta_2), \vartheta(\theta_1), \vartheta(w^{dist})\right)$
- $\vec{\chi}_{NB^r_{w_2}} = \left(\vartheta(w^{dist}), \vartheta(\kappa^f), \vartheta(\alpha), \vartheta(\tau_{e,f}), \mu_0^{(e)}, \vartheta(\epsilon), \vartheta(\theta_2), \vartheta(\theta_1)\right)$
- $\vec{\chi}_{NB^r_{w_3}} = \left(\vartheta(w^{dist}), \vartheta(\kappa^e), \vartheta(\alpha), \vartheta(\tau_{e,f}), \mu_0^{(e)}, \vartheta(\epsilon), \vartheta(\theta_2), \vartheta(\theta_1)\right)$
- $\vec{\chi}_{B^r_{rd}} = \left(\vartheta(\theta_1), \vartheta(q_e), \vartheta(\tau_e), \mu_0^{(e)}, \vartheta(\sigma_0^{(e)}), \vartheta(\epsilon), \vartheta(\theta_2)\right)$
- $\vec{\chi}_{B^r_{rw}} = \left(\vartheta(\kappa_e), \vartheta(q_e), \vartheta(\tau_e), \mu_0^{(e)}, \vartheta(\sigma_0^{(e)}), \vartheta(\epsilon), \vartheta(\theta_2), \vartheta(\theta_1)\right)$
- $\vec{\chi}_{B^r_d} = \left(\vartheta(q_{e,f}), \vartheta(\tau_{e,f}), \mu_0^{(e)}, \vartheta(\sigma_0^{(e,f)}), \vartheta(\epsilon), \vartheta(\theta_2), \vartheta(\theta_1), \vartheta(w^{dist})\right)$
- $\vec{\chi}_{B^f_d} = \left(\vartheta(q_{e,f}), \vartheta(\tau_{e,f}), \mu_0^{(e)}, \vartheta(\sigma_0^{(e,f)}), \vartheta(\epsilon), \vartheta(\theta_2), \vartheta(\theta_1), \vartheta(\theta_3), \vartheta(w^{dist})\right)$
- $\vec{\chi}_{B^r_{w_1}} = \left(\vartheta(\kappa_{e,f}), \vartheta(q_{e,f}), \vartheta(\tau_{e,f}), \mu_0^{(e)}, \vartheta(\sigma_0^{(e,f)}), \vartheta(\epsilon), \vartheta(\theta_2), \vartheta(\theta_1), \vartheta(w^{dist})\right)$
- $\vec{\chi}_{B^r_{w_2}} = \left(\vartheta(w^{dist}), \vartheta(\kappa_f), \vartheta(q_{e,f}), \vartheta(\tau_{e,f}), \mu_0^{(e)}, \vartheta(\sigma_0^{(e,f)}), \vartheta(\epsilon), \vartheta(\theta_2), \vartheta(\theta_1)\right)$
- $\vec{\chi}_{B^r_{w_3}} = \left(\vartheta(\kappa_f), \vartheta(w^{dist}), \vartheta(q_{e,f}), \vartheta(\tau_{e,f}), \mu_0^{(e)}, \vartheta(\sigma_0^{(e,f)}), \vartheta(\epsilon), \vartheta(\theta_2), \vartheta(\theta_1)\right)$
- $\vec{\chi}_{B^f_{w_1}} = \left(\vartheta(\kappa_{e,f}), \vartheta(q_{e,f}), \vartheta(\tau_{e,f}), \mu_0^{(e)}, \vartheta(\sigma_0^{(e,f)}), \vartheta(\epsilon), \vartheta(\theta_2), \vartheta(\theta_1), \vartheta(\theta_3), \vartheta(w^{dist})\right)$
- $\vec{\chi}_{B^f_{w_2}} = \left(\vartheta(w^{dist}), \vartheta(\kappa_f), \vartheta(q_{e,f}), \vartheta(\tau_{e,f}), \mu_0^{(e)}, \vartheta(\sigma_0^{(e,f)}), \vartheta(\epsilon), \vartheta(\theta_2), \vartheta(\theta_1), \vartheta(\theta_3)\right)$
- $\vec{\chi}_{B^f_{w_3}} = \left(\vartheta(\kappa_f), \vartheta(w^{dist}), \vartheta(q_{e,f}), \vartheta(\tau_{e,f}), \mu_0^{(e)}, \vartheta(\sigma_0^{(e,f)}), \vartheta(\epsilon), \vartheta(\theta_2), \vartheta(\theta_1), \vartheta(\theta_3)\right)$

where we used compressed notation, that is, $\vartheta(x_{a,b}) = (\vartheta(x_a), \vartheta(x_b))$ and $\vartheta(\vec{x}) = (\vartheta(x_1), \ldots, \vartheta(x_d))$

3 Model comparison

modelComparison*.mat: contains the outputs (posterior and out) of the function "VBA_groupBMC" of the toolbox for random-effect Bayesian model selection (RFX-BMS) at the group-level, which can be downloaded from toolbox for `https://sites.google.com/site/mbbvbtoolbox/download/RFX-BMS.zip`. Details on how to read the information provided in the output can be found either in the source code or at `https://sites.google.com/site/jeandaunizeauswebsite/code/rfx-bms`. The results of the analysis are shown in figure 10 of the main article.

The relevant data for the family-wise model comparison shown in figure 8 of the main article is saved familyComparison*.mat. The expected probability and the exceedance probability of the family-level model comparison are saved within variables 'out.families.Ef' and 'out.families.ep', respectively.

The accronim 'NSW' denotes the no-switch condition, 'SW' denotes the switch condition, and 'ALT' denotes the alternative perceptual model described in S1 Text.
