# Supplementary figures and images for: Modeling the Evolution of Beliefs Using an Attentional Focus Mechanism
Source: PLoS Comput Biol. 2015 Oct 23;11(10):e1004558. doi: 10.1371/journal.pcbi.1004558 (PMC4619749; doi:10.1371/journal.pcbi.1004558)

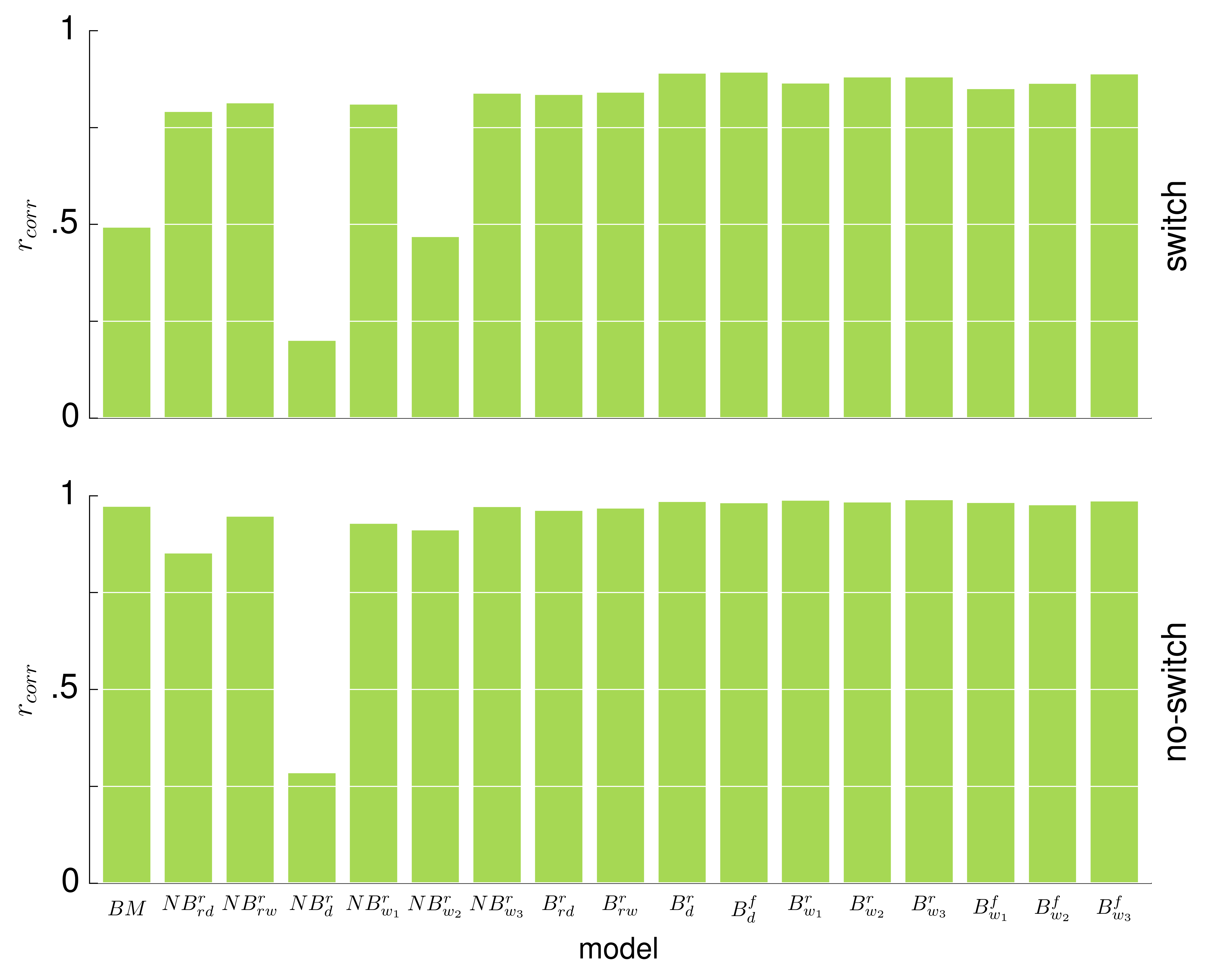

Supplement: S1 Fig — Pearson correlation coefficient r corr between the mean subject performance and the mean model performance, for each behavioral model in the switch (top) and no-switch condition (bottom). (TIFF) [file pcbi.1004558.s004.tiff]
